# The *Plasmodium vivax* Merozoite Surface Protein 3β Sequence Reveals Contrasting Parasite Populations in Southern and Northwestern Thailand

**DOI:** 10.1371/journal.pntd.0003336

**Published:** 2014-11-20

**Authors:** Chaturong Putaporntip, Jun Miao, Napaporn Kuamsab, Jetsumon Sattabongkot, Jeeraphat Sirichaisinthop, Somchai Jongwutiwes, Liwang Cui

**Affiliations:** 1 Molecular Biology of Malaria and Opportunistic Parasites Research Unit, Department of Parasitology, Faculty of Medicine, Chulalongkorn University, Bangkok, Thailand; 2 Department of Entomology, The Pennsylvania State University, State College, Pennsylvania, United States of America; 3 Vivax Malaria Research Center, Faculty of Tropical Medicine, Mahidol University, Bangkok, Thailand; 4 Vector Borne Disease Training Center, Pra Budhabat, Saraburi, Thailand; Arizona State University, United States of America

## Abstract

**Background:**

Malaria control efforts have a significant impact on the epidemiology and parasite population dynamics. In countries aiming for malaria elimination, malaria transmission may be restricted to limited transmission hot spots, where parasite populations may be isolated from each other and experience different selection forces. Here we aim to examine the *Plasmodium vivax* population divergence in geographically isolated transmission zones in Thailand.

**Methodology:**

We employed the *P. vivax* merozoite surface protein 3β (PvMSP3β) as a molecular marker for characterizing *P. vivax* populations based on the extensive diversity of this gene in Southeast Asian parasite populations. To examine two parasite populations with different transmission levels in Thailand, we obtained 45 *P. vivax* isolates from Tak Province, northwestern Thailand, where the annual parasite incidence (API) was more than 2%, and 28 isolates from Yala and Narathiwat Provinces, southern Thailand, where the API was less than 0.02%. We sequenced the *PvMSP3β* gene and examined its genetic diversity and molecular evolution between the parasite populations.

**Principal Findings:**

Of 58 isolates containing single *PvMSP3β* alleles, 31 sequence types were identified. The overall haplotype diversity was 0.77±0.06 and nucleotide diversity 0.0877±0.0054. The northwestern vivax malaria population exhibited extensive haplotype diversity (HD) of *PvMSP3β* (HD = 1.0). In contrast, the southern parasite population displayed a single *PvMSP3β* allele (HD = 0), suggesting a clonal population expansion. This result revealed that the extent of allelic diversity in *P. vivax* populations in Thailand varies among endemic areas.

**Conclusion:**

Malaria parasite populations in a given region may vary significantly in genetic diversity, which may be the result of control and influenced by the magnitude of malaria transmission intensity. This is an issue that should be taken into account for the implementation of *P. vivax* control measures such as drug policy and vaccine development.

## Author Summary

With intensified malaria control in endemic countries, there have been dramatic changes of malaria epidemiology. One of such changes is the increased proportion of *Plasmodium vivax* malaria, a demonstration of resilience of this parasite to control efforts. In Thailand, malaria has been largely eliminated from the central plain, and transmission is concentrated in isolated international border regions. This study aimed to examine whether the changing malaria epidemiology was reflected in the population dynamics and genetic diversity of the isolated parasite populations. We collected parasite samples from two regions in Thailand with drastically different endemicity settings and used a polymorphic genetic marker (*Plasmodium vivax* merozoite surface protein 3β – *pvmsp3β*) as an indicator of genetic diversity of the populations. Analysis of the *pvmsp3β* sequences revealed high genetic diversity of parasites from western Thailand, and suggested the suitability of this gene as a molecular marker to infer parasite genetic diversity. Comparing the *pvmsp3β* sequences, we further discovered extreme divergence in genetic diversity between the southern and northwestern Thai *P. vivax* populations. Our study offers important insights into malaria epidemiology and provides the needed knowledge for designing novel control tools in the malaria elimination campaigns.

## Introduction

Of the four species of human malaria parasites, *Plasmodium vivax* is the second most prevalent and the most geographically widespread parasite. Each year, *P. vivax* infects an estimated 130–391 million people, of which a large majority was in Central and Southeast Asia [1]–[3]. Recent data demonstrate that the traditionally called “benign tertian malaria” is certainly a misnomer since *P. vivax* infection brings enormous morbidity and mortality in affected populations [4], [5]. In addition, the development of resistance to chloroquine and possibly primaquine in *P. vivax* has raised a great concern for the control of the disease [6]. Outside sub-Saharan Africa, the proportions of malaria cases caused by *P. vivax* are arising, a clear indication of the resilience of this parasite to control measures [7]. Especially in areas of *P. falciparum* and *P. vivax* co-existence, their intricate interspecies interactions suggest that control measures against one species may inevitably lead to increased prevalence of the other [8], [9]. This has resulted in renewed interests in developing *P. vivax* vaccines. Vaccine development against such complicated eukaryotes like malaria parasites is not straightforward. Multivalent and multistage vaccines are proposed because the malaria parasite's life cycle involves multiple stages with each stage expressing different antigens. Merozoites as the invasive stage of the erythrocytic cycle are exposed to host immunity, and therefore are important vaccine targets [10]. Some merozoite antigens such as merozoite surface protein 1 (MSP1) and apical membrane antigen 1 (AMA1) have been extensively studied. Meanwhile, these antigens are subject to the selection forces imposed by the host immunity and exhibit extensive diversity [11]. As such, antigenic variation is an important consideration when identifying and prioritizing antigens for vaccine development.

A number of MSPs have been identified as the coat constituents of the *P. vivax* merozoites. These include PvMSP1 [12], the PvMSP3 family members [13], [14], PvMSP4, PvMSP5 [15], PvMSP7 [16], PvMSP8 [17], PvMSP9 [18], and PvMSP10 [19]. The first member of the PvMSP3 family identified in 1999 was named PvMSP3(α) due to its similarity to PfMSP3 [14]. Two paralogs were identified later and named PvMSP3β and 3γ, respectively [13]. In spite of limited sequence identify, the PvMSP3 protein family members share characteristics such as the central alanine-rich domain, which is predicted to form a coiled-coil structure that may involve in protein-protein interactions [13], [14]. Genome sequencing of the *P. vivax* Salvador I strain revealed 12 *PvMSP3* paralogs clustered in a ∼60 kb locus on chromosome 10, which led the authors to speculate that this gene family might have undergone species-specific expansion [20]. Although a number of studies suggested relatedness of *msp3* genes in *P. vivax* and *P. falciparum*, a closer comparison between the syntenic loci on chromosome 10 and domain organizations of *pvmsp3* and *pfmsp3* did not suggest that these are homologs [21]. Though these putative msp3 family proteins share an N-terminal NLRNG peptide motif, comparison of the *msp3* loci in several *Plasmodium* species of Asian primates and the African monkey parasite *Plasmodium gonderi* further established that the expanded *pvmsp3* family had a much earlier origin. Furthermore, analysis of several additional *P. vivax* genomes found expansion and contraction of the *pvmsp3* loci, with *pvmsp3* family members ranging from 9 to 14 in each parasite genome [21]. Based on this finding, the authors hypothesized that the *pvmsp3* gene family might be under multi-allelic diversifying selection to increase antigenic diversity [21].

Recent studies detected protein expression for 10 members of the PvMSP3 family with eight proteins being visualized surrounding merozoites and one being localized at the apical end of merozoites [22]. Since PvMSP3 family members do not appear to have transmembrane domains or the GPI-anchor site, their association with the merozoite surface is predicted to be through protein-protein interactions via the central coiled-coil domain [13], [14]. Yet, this region in both PvMSP3α and PvMSP3β harbors large deletions in worldwide collections of *P. vivax* strains [23]–[26]. Sequencing analysis revealed that both genes are highly polymorphic and the highest nucleotide diversity is clustered towards the N-terminal region [26]–[34], making them very useful genotyping markers for differentiating *P. vivax* field isolates. PCR-RFLP methods developed for genotyping *pvmsp3α* and *3β* have been used widely for studying *P. vivax* genetic diversity in various endemic settings [29], [30], [33], [35]–[39], albeit caution needs to be exercised when interpreting the RFLP patterns since frequent insertion-deletion mutations and recurrent recombination events may obscure the distinctions between RFLP haplotypes [21]. Despite their polymorphic nature, the potential of PvMSP3α and 3β as vaccine candidates has been evaluated. Both proteins are found immunogenic and naturally acquired antibodies are associated with exposure to *P. vivax* parasites in vivax-endemic regions [40]–[43]. Antibodies against PvMSP3α in Papua New Guinea children were associated with protection from clinical *P. vivax* malaria [44]. Therefore, further evaluation of the polymorphism of these proteins in endemic countries is needed.

In this study, we have obtained full-length *PvMSP3β* gene sequences from 58 *P. vivax* clinical samples collected mostly from two endemic regions in Thailand. This has allowed us to further evaluate the genetic diversity and dissect the domain structure of PvMSP3β. We demonstrate drastic geographical differentiation of *P. vivax* populations, with its genetic structure being correlated with the endemicity of *P. vivax* malaria. The monomorphic *pvmsp3β* gene in a *P. vivax* population from the hypoendemic southern Thailand suggests a clonal expansion of the parasite strain.

## Materials and Methods

### Sources of parasite isolates

*Plasmodium vivax* isolates were collected from two study sites in northwestern and southern Thailand. Forty-five patients were recruited at the malaria clinic located in Mae Sot district, Tak province, northwestern Thailand [31], while 28 isolates were from Yala and Narathiwat Provinces, southern Thailand [45]. Collection of finger-prick filter paper samples from malaria patients was approved by the institutional review board of Chulalongkorn University under the auspice of the Thai Ministry of Health. After obtaining written informed consent, blood samples were collected on filter papers and dried. Parasite DNA was extracted from the filter papers using a QIAamp DNA Mini kit (Qiagen, Germany) and DNA was eluted in 100 µl of water.

### Polymerase chain reaction (PCR), cloning, sequencing, sequence assembly

The complete *pvmsp3β* gene of 2.0–2.5 kb was amplified using primers Pv3BF (5′ AAATGGTATTCTTCGCAACAC 3′) and Pv3BR (5′ TTCGTCACCAATTTGTTTAGC 3′). All primers were designed based on the PvMSP3β gene (AF099662) of the Belem strain [13]. PCR was done in 25 µl consisting parasite DNA, 1× reaction buffer, 200 µM of dNTPs, 0.05 µg each of the outer primers, and 0.5 µl of KlenTaq (BD Biosciences) using a program of 1.5 min of initial denaturing at 94°C followed by 35 cycles of 94°C for 30 sec, 55°C for 30 sec and 68°C for 3 min. The PCR products were sequenced directly using the BigDye terminator kit (Applied Biosystems). For accuracy, two different amplification products of each isolate were sequenced. Complete sequences were assembled using the DNASTAR program (Lasergene). Both DNA and predicted protein sequences were aligned using Clustal X version 1.83. The internal region of the sequences was further edited manually due to the presence of insertion/deletion in several isolates. Sequences available in the GenBank that were included in this analysis are from isolates/strains from Bangladesh (AY454084), Brazil (PVBG05499, AF099662, AY454080, AY454081, AY454082, AY454085, AY454086, AY454087, AY454088, AY454089), El Salvador (XM001613146), Ecuador (AY454091), India (PVIIG04181, AY454092), Mauritania (PVMG01384), Papua New Guinea (AY454083), North Korea (PVNG01493), Sri Lanka (AY454096 and AY454097), Thailand (AY454098) and Vietnam (AY454094). New *pvmsp3β* sequences in this study were submitted to GenBank (KM041050 to KM041113).

### Genetic diversity, selection, and recombination

Molecular evolutionary analysis was performed using MEGA version 6.0 [46] and DnaSP version 5.10.1 [47]. The genetic diversity of *pvmsp3β* gene was estimated using the parameter π, which calculates the average number of substitutions per site between two sequences. The rates of synonymous (*d*_S_) and nonsynonymous (*d*_N_) substitutions were estimated using the method of Nei and Gojobori with the Jukes and Cantor correction. The standard error was calculated using the bootstrap method with 1000 pseudoreplications. To test whether *pvmsp3β* is under positive selection, Z-test was performed by comparing the nonsynonymous and synonymous substitutions using MEGA. Different regions of the gene were further evaluated by a sliding window analysis and their significance was determined using the Tajima's D test [48] and the D* and F* statistics of Fu and Li [49]. The minimum number of recombination events (Rm) was estimated using DnaSP. The degree of linkage disequilibrium (LD) between distance of parsimony informative variant nucleotide sites was estimated by the *r^2^* values [47] and significance levels (more than 95%) were determined by the two-tailed Fisher's exact test. Evidence of intragenic recombination was also determined by using the RDP4 package [50] and the Genetic Algorithm Recombination Detection (GARD) method implemented in the HyPhy package [51].

### Phylogenetic analysis

The evolutionary relationships of the *P. vivax* isolates were inferred from phylogenetic analysis of the complete or available *pvmsp3β* gene using the Maximum Likelihood method based on the General Time Reversible model. The Neighbor-Joining method was applied to generate initial trees for the heuristic search using the Maximum Composite Likelihood approach with a discrete Gamma distribution to model evolutionary rate differences among sites. The reliability of the tree was assessed by the bootstrap method with 500 pseudoreplications. The analysis was performed using the MEGA 6.0 [52].

### Genetic structure

The population genetic structure was evaluated by the analysis of molecular variance (AMOVA) approach implemented in the Arlequin 3.5 software [53] using the Weir and Cockerham's method while taking into account the number of mutations between haplotypes [54]. All domains were included in the analysis excluding sites with alignment gaps. Populations were classified as South America (n = 13), Asia excluding Thailand (n = 13) and Thailand (n = 59). The fixation index *F_ST_* identical to the weighted average *F*-statistic over loci, θw [54] and the significance levels of the fixation indices were estimated by non-parametric permutation as implemented in Arlequin 3.5 [53].

## Results

### Drastic regional difference in *pvmsp3β* diversity

We have amplified and sequenced a total of 73 *P. vivax* clinical samples from two study sites in Thailand. The PCR products were sequenced directly. Mixed infections were evidenced by the presence of multiple PCR bands and superimposed signals on electropherogram from DNA sequencing. All 28 samples from southern Thailand were single clone infections. However, 15 of the 45 samples (33%) from Mae Sot in northwestern Thailand had multiple clone infections and were excluded from sequencing analysis. Four isolates from China and one isolate from India were sequenced and included in this study. The size of the *pvmsp3β* sequences ranges from 2,109 to 2,478 bp, encoding a predicted protein of 703–826 amino acids (aa). Our data revealed drastic difference in haplotype diversity of *pvmsp3β* gene between the two study sites. The 30 *pvmsp3β* sequences from northwestern Thailand are all unique (haplotype diversity = 1). Surprisingly, 28 sequences from southern Thai *P. vivax* population were all identical, suggesting a potential result of clonal expansion.

### Domain organization and genetic diversity of *PvMSP3β* gene

**Figure 1:**
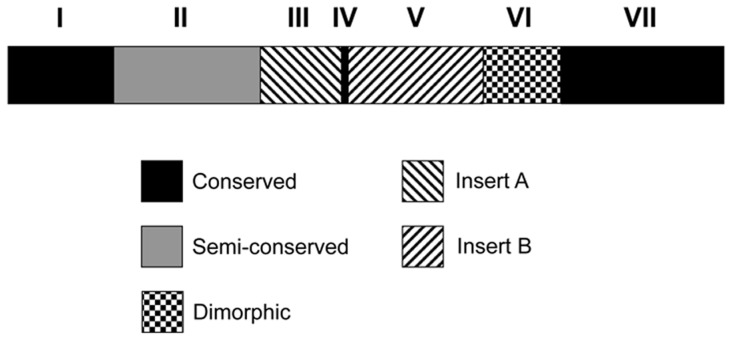
Domain organization of *PvMsp3β*. Based on sequence conservation, the *PvMsp3β* was divided into 7 blocks: conserved Block 1 (1–471), semiconserved Block 2 (472–1008), insert A –Block 3 (1009–1368), conserved Block 4 (1369–1392), Insert B – Block 5 (1393–1992), dimorphic Block 6 (1993–2331) with the Sal-1 and Bangl Type, and conserved Block 7 (2332–2901). The numbers in parentheses are range of the nucleotide sequences from the Salvador-1 strain.

**Figure 2:**
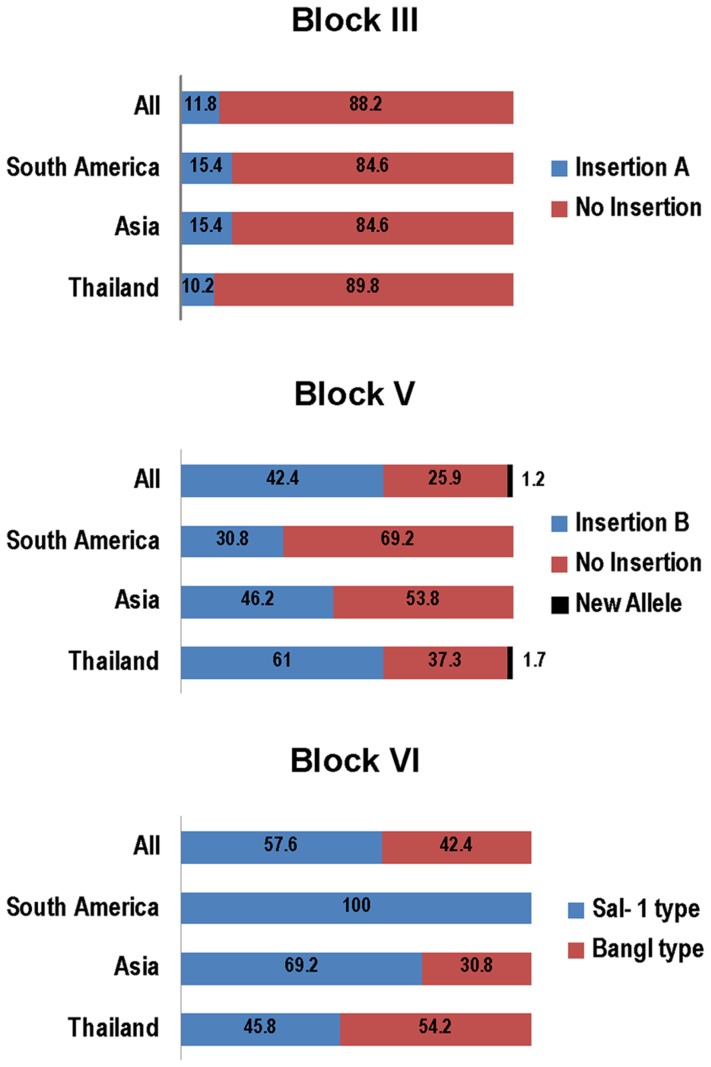
Allelic distribution of blocks 3, 5 and 6 of *PvMSP3β* among isolates from South America (n = 13), Thailand (n = 59) and Asia excluding Thailand (n = 13) shown as a percentage.

Previous studies showed that *pvmsp3β* gene has large variations in gene size among world parasite populations [26], [33], [34], which are due to insertions or deletions occurring in the central domain of the gene. Alignment of the predicted aa sequences from Thailand and those determined earlier [26] showed that PvMSP3β is highly polymorphic, containing numerous substitutions, insertions and deletions of variable sizes (Figure S1). The distribution of substitutions is not random, and the C-terminal block is more conserved. Most notable is the two blocks of large insertions in the central Ala-rich domain. Previous report by Rayner *et al.* (2004) divided the gene into four regions: the N-terminal part, insert A, insert B and the C-terminal part. Based on more detailed analysis of available sequences, we divided the sequences into seven blocks. Figure 1 is a schematic representation of the PvMSP3β protein using the Salvador I (Sal-1) strain as the reference. Block 1 (1–157 aa) is the conserved N-terminus without obvious insertions or deletions. Block 2 (158–336 aa) is less conserved with multiple interspersed short insertions or deletions, which is followed by insertion A (337–456 aa) referred here as block 3. Block 4 is conserved, spanning a short region of 24 nucleotides (457–464 aa). Insertion B or block 5 (465–664 aa) is highly polymorphic, and the Thai isolate-105 has a novel sequence in this block. Block 6 (665–777 aa) was originally included in a portion of insertion B, which is a dimorphic region represented by the Sal-1 type and the Bangl type. Block 7 (778–967 aa) is the conserved C-terminus. BR67B is the only isolate that has an unusually long deletion in this region [26]. Worldwide distribution of *pvmsp3β* alleles based on blocks 3, 5 and 6 is shown in Figure 2. Most of the sequences (89%) did not harbor insertion A. Samples with insertion B of block 5 was less prevalent. Most of the sequences in the dimorphic C-terminal block 6 had the Sal-1 type, whereas the Bangl type was absent in the American samples.

The overall nucleotide diversity of *pvmsp3β* was similar to the estimates for other *P. vivax* merozoite antigens such as *pvmsp1*, *pvama1* and *pvmsp3α* [23], [55]. The conserved C-terminal block was the least variable, whereas the N-terminal regions had more than two times higher nucleotide diversity (Table 1).

**Table 1:** Patterns of nucleotide substitutions in *PvMsp3β* from each endemic region.

Block	*P. vivax*	n	nt	H	S(N)	π ± S.D.	*h* ± S.D.	*d*_S_ ± S.E.	*d*_N_ ± S.E.	Tajima's *D*	Fu & Li's	
											*D* *	*F* *
1 (Conserved)	Thailand	59	351	30	120(134)	0.0770±0.0058	0.778±0.060	0.0622±0.0153	0.1060±0.0126*	−0.38	−1.191	−0.976
	Asia#	13	351	12	80(86)	0.0870±0.0050	0.987±0.001	0.0481±0.0140	0.1072±0.0143**	−0.954	0.614	0.657
	America	12	351	8	59(63)	0.0605±0.0108	0.894±0.078	0.0490±0.0187	0.0872±0.0127	0.137	0.2	0.192
	All	84	351	49	125(141)	0.0830±0.0032	0.888±0.033	0.0571±0.0141	0.1050±0.0130*	−1.131	−1.055	−0.682
2 (Semi-conserved)	Thailand	59	474	31	228(265)	0.0993±0.0082	0.778±0.060	0.1119±0.0184	0.1487±0.0131	0.136	−1.707	−1.534
	Asia	13	477	12	169(194)	0.1338±0.0084	0.987±0.035	0.1225±0.0221	0.1580±0.0145	0.468	0.168	0.17
	America	12	486	8	132(140)	0.1016±0.0187	0.924±0.057	0.0692±0.0167	0.1196±0.0116*	0.81	1.115	1.028
	All	84	474	44	228(269)	0.1130±0.0049	0.878±0.034	0.1084±0.0185	0.1480±0.0128	−0.809	−1.087	−0.76
3 (Insert A)	All	10	360	7	78(80)	0.0729±0.0232	0.911±0.077	-	-	-	-	-
4 (Conserved II)	Thailand	59	24	6	9(13)	0.0625±0.0117	0.653±0.054	0.3751±0.1986	0.2075±0.0898	0.385	0.097	0.221
	Asia	13	24	6	4(5)	0.0684±0.0089	0.833±0.081	0.0916±0.0901	0.0868±0.0449	0.066	0.532	0.468
	America	12	24	5	4(4)	0.0562±0.0117	0.742±0.116	0.0560±0.0743	0.1010±0.0529	0.064	−0.458	−0.369
	All	84	24	8	9(14)	0.0660±0.0081	0.754±0.030	0.3359±0.1662	0.1733±0.0799	0.408	0.202	0.318
5 (Insert B)	All	47	234	13	164(199)	0.1493±0.0272	0.510±0.090	-	-	-	-	-
6 (Dimorphic) Sal-1	Thailand	27	333	25	87(93)	0.0594±0.0045	0.994±0.012	0.0855±0.0184	0.0585±0.0087	−0.698	−1.487	−1.451
	Asia	9	339	7	54(57)	0.0601±0.0085	0.944±0.070	0.0874±0.0220	0.0660±0.1070	-	-	-
	America	12	273	6	47(52)	0.0601±0.0084	0.848±0.074	0.1046±0.0235	0.0611±0.0110	−0.220	−0.193	−0.228
	All	48	267	31	84(92)	0.0646±0.0042	0.962±0.016	0.0909±0.0195	0.0588±0.0084	−0.598	−1.166	−1.512
Bangl	Thailand	32	342	3	18(18)	0.0033±0.0027	0.123±0.078	0.0524±0.0222	0.0319±0.0101	−2.545***	−4.656*	−4.682*
	Asia	4	342	2	13(13)	0.0253±0.0077	0.667±0.204	0.0425±0.0249	0.0381±0.0123	-	-	-
	America	-	-	-	-	-	-	-	-	-	-	-
	All	36	342	3	15(15)	0.0046±0.0027	0.165±0.082	0.0613±0.0205	0.0406±0.0102	−2.298*	−2.151	−2.603*
7 (Conserved)	Thailand	59	570	28	90(97)	0.0331±0.0026	0.728±0.066	0.3068±0.0630	0.1881±0.0191	−0.338	−1.720	−1.423
	Asia	13	570	12	55(58)	0.0334±0.0035	0.987±0.035	0.5144±0.1286	0.3092±0.0354	0.084	0.429	0.385
	America	11¶	570	7	47(48)	0.0334±0.0023	0.909±0.066	0.1258±0.0325	0.0606±0.0136	0.587	1.195	1.177
	All	84¶	570	42	99(08)	0.0365±0.0012	0.857±0.038	0.6303±0.1438	0.3535±0.0435	1.025	−0.299	0.177

# Excluding Thailand.

¶ Excluding Br67B.

n, number of sequence; nt, number of nucleotide; H, number of haplotype; S, Number of segregating site; N, Number of mutation; *h*, haplotype diversity; π, nucleotide diversity; *d*_S_, mean values of synonymous substitutions per synonymous site and *d*_N_, mean values of nonsynonymous substitutions per nonsynonymous site.

*, ** and *** are significance at the 5, 0.5 and 0.05% levels, respectively.

### Evidence of selection

The PvMSP proteins harbor extensive sequence diversity in the N-terminal region, suggesting of selection. To identify the signature of selection in *pvmsp3β* in the parasite populations, we performed neutrality tests on individual blocks of *pvmsp3β* where nucleotide sequences could be unambiguously aligned. Block-wise analysis showed that *d*_N_ is significantly greater than *d*_S_ in blocks 1 and 2, suggesting positive selection in these blocks. In contrast, *d*_S_ significantly out-numbers *d*_N_ in block 7, suggesting purifying selection in the C-terminal-coding region. Although *d*_S_ was greater than *d*_N_ in most of blocks 4 and 6, they were not significantly different. Tajima's *D* statistics, however, did not detect signatures of selection in block-wise analysis (Table 1). On the other hand, significant departure from neutrality was observed in Fu and Li's D* and F* tests for conserved block 4 and isolates harboring Bangl type of the dimorphic block 6. Negative departure from neutrality of these tests could be resulted from purifying selection or population expansion after a bottleneck effect.

### Intragenic recombination

**Figure 3:**
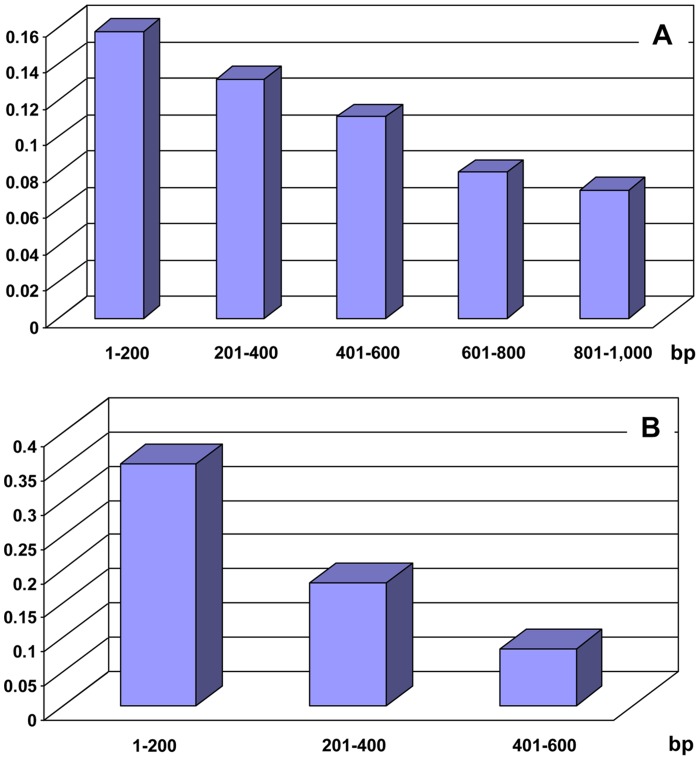
Proportions of significant linkage disequilibrium between polymorphic sites within blocks 1, 2 and 4 (A) and within block 7 (B) of PvMSP3*β* in *P. vivax* population from Thailand. X axis indicates molecular distance between polymorphic informative loci. Y axis is the *r^2^* value from LD analysis.

The minimum number of recombination events (Rm) estimated using DnaSP showed that for the Thai isolates 72 recombination events were detected in blocks 1, 2 and 4. The high number of predicted Rm value might be accountable for the observed slight decay in significant loci over molecular distance within blocks 1, 2 and 4 as revealed from LD analysis (Figure 3A). Likewise, decay in significant loci over molecular distance was also observed in block 7 although relatively lower Rm value of 17 was identified in this block (Figure 3B). Analysis of recombination break points inferred 40 recombination events by one or more methods implemented in the RDP4 program package (RDP, GENECONV, BOOTSCAN, MAXCHI, CHIMERA, SISCAN, 3SEQ, PHYLPRO and LARD) using the default parameters (Figure S2). Additional analysis using the GARD program implemented in the HyPhy package revealed that at least 3 break points (nucleotides 406, 1500 and 2608, positions based on the alignment shown in Figure S1) gave significant topological incongruence (p<0.01) between AICc (Akaike Information Criterion derived from a maximum likelihood model fit to each segment) score of the best fitting GARD model.

### Phylogenetic analysis

**Figure 4:**
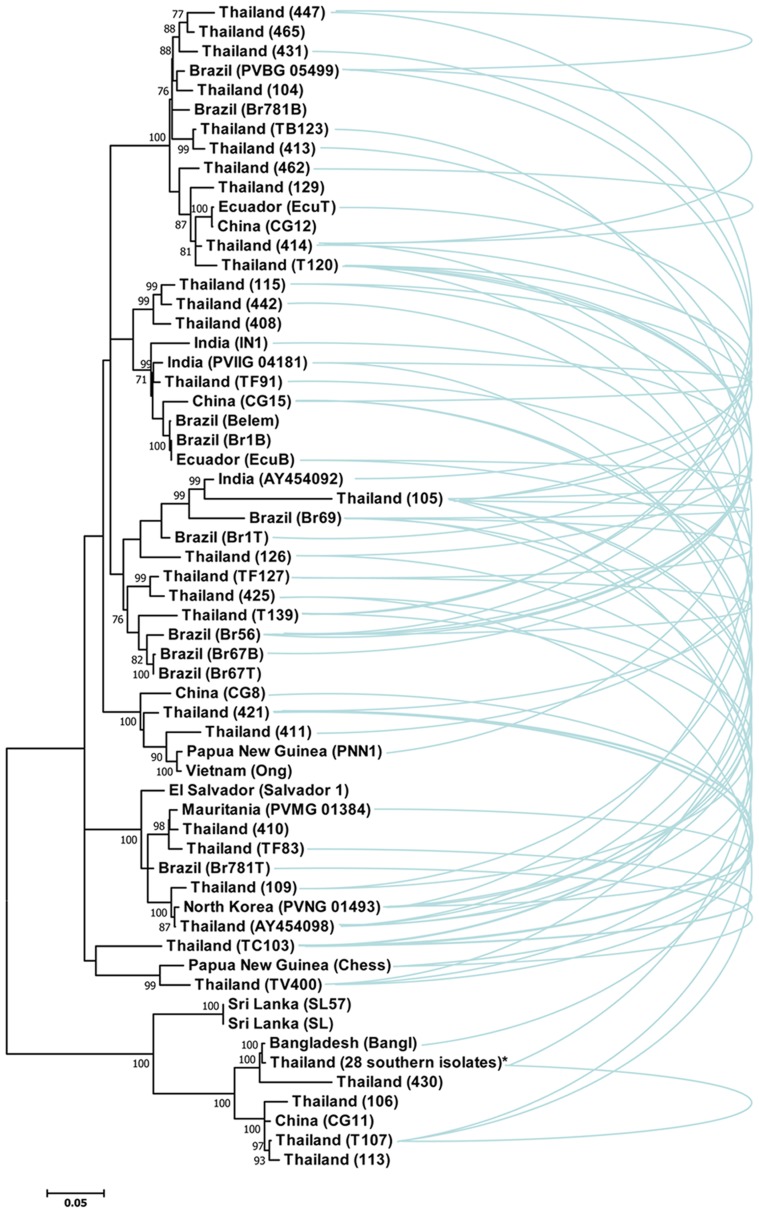
Phylogenetic tree inferred from aligned positions of *PvMSP3β* using the Maximum Likelihood method based on the General Time Reversible model. The tree with the highest log likelihood (−19878) is shown. The tree is drawn to scale, with branch lengths measured in the number of substitutions per site. The bootstrap values greater than 75% are shown along the branch. Isolate names (or accession numbers) are listed in parentheses after their geographic origins. “28 southern isolates” are those from Yala (n = 9) and Narathiwat Provinces (n = 19). Recombination events between sequences based on the RDP4 algorithms are shown as blue curved lines connecting between isolates.

We used the coding region of PvMSP3β gene to infer the phylogenetic relationship among the isolates. Phylogenetic analysis revealed no geographic clustering among isolates from diverse origins and that Thai alleles were placed throughout the tree (Figure 4). This phenomenon has also been observed for the PvMSP3α gene among the Thai isolates [23]. However, phylogenetic tree has defined two distinct clusters of isolates with 100% bootstrap values, corresponding to the dimorphic Sal-1 type and the Bangl type (Figure 4). It is noteworthy that recombination events inferred from the RDP4 algorithm could be commonly observed between isolates within and between diverse geographic origins (Figure 4) akin to those occurred at the *pvmsp3α* locus [23].

### Genetic structure

Despite the small number of samples used in this study, we observed significant genetic structure among isolates from Thailand (n = 59), Asia excluding Thailand (n = 13) and the Americas (n = 13). The highest *F_ST_* value was noted between *P. vivax* populations from Thailand and the Americas (Table 2).

**Table 2:** Genetic differentiation (*F*_ST_ indices, lower diagonal) and significance level (*p* values, upper diagonal) of *P. vivax* populations.

	Thailand	Asia#	America
Thailand		0.02703	0.00000
Asia#	0.09913		0.03604
America	0.28128	0.09935	

# Excluding Thailand.

## Discussion

The contemporary malaria parasite populations have been shaped by many selective forces such as human malaria control activities. As the malaria control and elimination course progresses, it is expected that the range of malaria endemicity will shrink, which should influence the effective population size. In many countries of the Greater Mekong Subregion (GMS) of Southeast Asia, malaria is restricted to the international border regions and some even exists in isolated pockets, further restricting gene flow among parasite populations. In this study, we investigated the genetic diversity and evolution of the *pvmsp3β* gene in parasites from two endemic regions of Thailand. We have further confirmed that sequencing of *pvmsp3β* offers significantly increased power for determining parasite genetic diversity compared with the simple PCR-RFLP method [33]. This is analogous to using *pvmsp3α* as a molecular marker, where PCR-RFLP is not as informative of population genetic diversity [32]. In the northwestern Thai provinces bordering Myanmar, the progress of malaria control has been slow, and cross-border human population movements and malaria introduction from Myanmar have been partially blamed for the continued malaria transmission [56]. Our analysis of the PvMSP3β polymorphism revealed that *P. vivax* parasites originated from the northwestern border region were extremely diverse, a result that is consistent with the analysis of PvMSP3α gene from the same parasite population [23], [31]. The *P. vivax* parasite population has attained a haplotype diversity approaching 1 at both loci. Our finding of extensive diversity of the vivax parasite population in the western border of Thailand is in line with the results from genotyping other genetic loci such as PvMSP1 and PvAMA1 [57].

Despite the low level of malaria transmission in this region, genotyping PvMSP3α and PvMSP3β loci consistently revealed that more than 30% of vivax patients still harbored mixed strain infections [58]. Mixed parasite infection may have resulted from activation of latent heterologous hypnozoites in the liver by new parasite infections [59], . Inoculation by the same mosquito carrying mixed parasite strains [61] or by different mosquitoes carrying different parasite strains may play an additional role. Mixed-strain infections favor genetic recombination, generation of new genetic alleles, and maintenance of genetic diversity. In agreement, analysis of intragenic recombination revealed numerous number of recombination events within the *PvMSP3β* gene. Ultimately, high genetic recombination rates correspond to the high genetic diversity observed in western Thailand. Since the levels of genetic diversity and endemicity are often correlated [62], [63], the high genetic diversity observed with the *PvMSP3β* gene may indicate higher malaria endemicity at the Thai-Myanmar border area.

Using the *PvMSP3β* gene as a molecular marker, we have detected significant parasite population differentiation. While it is easy to understand that the Thai and American parasite populations differ drastically because of geographical separation (*F*st = 0.28), we also observed significant population structure of the parasites from the GMS. While this result supports those derived from analysis of other polymorphic markers, such studies will need to be bolstered by using larger sample sizes and multiple molecular markers.

Our block-wise analysis has shown significant negative departure from neutrality of Tajima's *D*, Fu and Li's D* and F* values for the dimorphic Bangl type of block 5, which mostly include isolates from southern Thailand. This seems to be due to population expansion after bottleneck effect rather than strong purifying selection because the rates of nucleotide substitutions at synonymous and nonsynonymous sites did not differ significantly (Table 1). In the extreme south that borders Malaysia, control activities since the early 1990s have led to a sharp reduction of the annual malaria incidence. As a result, the parasite genetic diversity has also been curtailed. Genotyping multiple merozoite antigens from the southern parasite population revealed drastically reduced haplotype diversity [57]. While reduced genetic diversity of the malaria parasite populations due to intensified control efforts has been reported for *P. falciparum* parasites [64]–[66], *P. vivax* populations are more resilient to control measures. For example, in the Solomon Islands which is progressing towards malaria elimination, despite that the *P. falciparum* population exhibited low diversity, *P. vivax* populations remained highly diverse as revealed by microsatellite genotyping [63]. Similarly, in the temperate zone region of central China, *P. vivax* parasites also maintained relatively high genetic diversity as shown for the *pvmsp3β* diversity in spite of drastic reduction in annual incidence [34]. In this regard, our finding of a single haplotype from 28 southern Thai parasite samples suggestive of clonal expansion is truly surprising. Without a potential cause of selective sweeps such as drugs, mosquitoes or host immunity that might have led to the sharp decline of genetic diversity in this region, a bottleneck hypothesis is very plausible [57]. Under this scenario, extensive malaria control efforts and lack of re-introduction from the neighboring country dramatically reduced the parasite population size, resulting in the survival of only a small number of parasite strains. In recent years, political unrest and the consequential inability to deploy effective control measures in the southern region have led to an increase in malaria incidence during the past decade. Since the majority of the cases in the southern region were indigenous, they were highly likely resulting from clonal expansion of the remaining parasite strains. This suggestion based on the analysis of *pvmsp3β* is supported from the analysis of another highly polymorphic marker PvAMA1 [57]. This analysis also highlights the need of more genetic markers to infer population structure of the parasites. These findings suggest that initial non-vaccine control measures leading to severe parasite population bottlenecks may circumvent the problem associated with antigen polymorphism in vaccine development.

The high level of genetic polymorphism of vaccine candidates poses a major challenge for malaria vaccine development. Since effective immunity against these vaccine candidates are often strain-transcending, meaning that antibodies against one strain may not be effective against another, the genetic diversity of a vaccine candidate needs to be evaluated in regions of potential deployment. Many of the MSP proteins contain highly polymorphic and conserved domains, signifying the results of diversifying selection and functional constraints, respectively. The most studied PvMSP1 has a mosaic structure composed of seven conserved and six variable blocks. The potentially high frequencies of recombination suggested from the presence of numerous recombination sites within the locus may be responsible for the observed linkage equilibrium at the distance of>3 kb [55]. In the case of *pvmsp3β*, we identified a remarkably high level of nucleotide diversity, especially in the 5′ half of the gene. This genetic diversity might have been maintained by balancing selection and reinforced by frequent genetic recombination, which is supported by the high Rm values, and identification of decay of LD with the increase of molecular distance in the N-terminal region. This finding together with our earlier study of PvMSP3α suggests that PvMSP3 family proteins share a similar domain structure with N-terminal regions exposed on the merozoite surface, which are subject to strong balancing selection by host immunity. This has potential implications for further considering PvMSP3β's vaccine potential, as antibodies against such domains are often allele-specific [67]. The high level of genetic diversity in the N-terminal region argues against consideration for vaccine development, whereas the relatively conserved C-terminal region may be more favorable. In endemic area of Brazil, a large proportion of vivax-infected individuals developed antibodies against PvMSP3α and PvMSP3β, indicating immunogenicity of both proteins [42]. As speculated, significantly more people contained antibodies recognizing the C-terminal portion of the PvMSP3β protein [42]. Since the PvMSP3 gene family in a parasite strain can contain more than 12 paralogs and they potentially have redundant roles [21], [22], evaluation of the vaccine potential for conserved members is needed.

## Supporting Information

Figure S1 Alignment of deduced amino acid sequences of *PvMSP3β* of Thai isolates and those available in the GenBank database. Gap and asterisk represent deletion and missing data, respectively. Positions are shown on the right margin of each alignment. Block boundary is marked by > and <. Isolates from Yala (n = 9) and Narathiwat Provinces (n = 19) are identical and only representative isolate NR1 is shown.
(DOCX)

Figure S2 Recombination events and breakpoints in the *PvMSP3β* locus determined from the RDP4 package. Recombination fragments are shown as bars below the gene scheme depicted after Figure 1. Scale is for aligned nucleotide sites.
(PDF)
