# Supplementary material for: The Plasmodium vivax Merozoite Surface Protein 3β Sequence Reveals Contrasting Parasite Populations in Southern and Northwestern Thailand
Source: PLoS Negl Trop Dis. 2014 Nov 20;8(11):e3336. doi: 10.1371/journal.pntd.0003336 (PMC4238993; doi:10.1371/journal.pntd.0003336)
Supplement: Figure S1 — Alignment of deduced amino acid sequences of PvMSP3β of Thai isolates and those available in the GenBank database. Gap and asterisk represent deletion and missing data, respectively. Positions are shown on the right margin of each alignment. Block boundary is marked by > and <. Isolates from Yala (n = 9) and Narathiwat Provinces (n = 19) are identical and only representative isolate NR1 is shown. (DOCX) [file pntd.0003336.s001.docx]

< Conserved

#XM001613146-Sal MKQFCGLAFL ALLLNFLTCD NVATRGEIVN LKNPNLRNGW SMKNLSAQNE ENIVHSDGSD DVTDKEEDGE VLEGQKGSPK KSAEQKVHAQ EEVNKESLKS KAQNAKAEAE KAAKAAESAK [ 120]

#AY454081-Br1T MKQFCGLAFL ALLLNFLTCD NAATRGEIVN LKNPNLRNGW SMNNLSAQNE ENIVNPDGSD DVTDEEGDGE ALEGQNGSPE KSAEPKVHAQ EEVNKESLKS KAQNAKAEAE KAAKAAESAK [ 120]

#AY454082-Br69 MKQFCGLAFL ALLLNFLTRD NVATRGEIVN LKNPNLRNGW SMKNLSAQNE ENIVNSHGSD DVTDKKEDGE VLEGQKGSPK KLAEPKVHAQ EAETKESLKS KAQNAKAEAE KAAKAAESAK [ 120]

#AY454092-India ********** ********** ********** ********** SMNNLSAQNE ENIVNPDGSD DVTDEEGDGE VLEGQNGSPE KSAEPKVHAQ EEETKESLKS KAQNAKEEAE KAAKAAASAK [ 120]

#AY454091-EcuT ********** ********** ********** ********** SMKNLSAQNE ENIVNPDGSD DVTDEEGDGE ALEGQKGSPK KSAEPKVHAQ EEGNKDELKS KATKAKADAT EAVKAAELAK [ 120]

#Thai-414 MKQFCGLAFL ALLLNFLTCD NVATRGEIVN LKNPYLRNGW SMNNLSAQNE ENIVHSDGSD DVTDKEEDGE VLEGENESPK KSAEPKVHAQ EAESKESLKL KAANAKKDAE EAAKAAQSAK [ 120]

#Thai-129 MKQFCGLASL ALLLNFLTCD NVATRGEIVN LKNTNLRNGW SMKNLSAQNE ENIVHSDGSD DVTDEEGDGE VLEGQNGSPE KSAEPKVHAQ EEETKESLKL KAANAKKDAE EAAKAAQSAK [ 120]

#Thai-T120 MKQLCGLAFL ALLLNFLTCD NVATRGEIVN LKNPNLRNGW SMKNLSAQNE ENIVNSHGSD DVTDKKEDGE ALEGQNGSPK KSAEPKVHAQ EAETKESLKL KAANAKKDAE EAAKAAQSAK [ 120]

#Thai-462 MKQLCGLAFL ALLLNFLTRD NVATRGEIVN LKNTNLRNGW SMKNVSAQNE ENIVHPDGSD DVTDKEEDGE VLEGENESPK RSAEPKVHAQ EAESKESLKL KAANAKKDAE EAAKAAQSAK [ 120]

#China-CG12 MKQLCGLAFL ALLLNFLTRD NVATRGEIVN LKNPYLRNGW SMKNLSAQNE ENIVNPDGSD DVTDEEGDGE ALEGQKGSPK KSAEPKVHAQ EEGNKDELKS KATKAKADAT EAVKAAELAK [ 120]

#China-CG8 MKQLCGLAFL ALLLNFLTCD NVATRGEIVN LKNTNLRNGW SMNNLSAQNE ENIVNSHGSD DVTDKEENGE VLEGENGSPK KLAEPKVHAQ EAETKESLKL KATKAKTEAV EAANAAELAK [ 120]

#AY454084-Bangl ********** ********** ********** ********** SMKNVSAQNE ENIVHSDGSD DVTDKEEDGE VLEGQKGSPK KSAEPKVHAQ EEGNKDELKS KATKAKADAT EAVKAAELAK [ 120]

#AY454097-SL57 ********** ********** ********** ********** SMKNVSAQNE ENIVHLDGSD DVTDKEEDGE VLEGQNGSPK KLAEPKVHAQ EEETKDELKS KATKAKTEAV EAAKAAETAK [ 120]

#AY454096-SL ********** ********** ********** ********** SMKNVSAQNE ENIVHLDGSD DVTDKEEDGE VLEGQNGSPK KLAEPKVHAQ EEETKDELKS KATKAKTEAV EAAKAAETAK [ 120]

#Thai-106 MKQLCGLAFL ALLLNFLTCD NVATRGEIVN LKNPNLRNGW SMNNLSAQNE ENIVNSHGSD DATDKEEDGE VLEGENESPK KLAEPKVHAQ EVVNKESLKS KATTAKTEAE KAANAADLAK [ 120]

#Thai-NR1 MKQLCGLAFL ALLLNFLTCD NVATRGEIVN LKNPNLRNGW SMKNLSAQNE ENIVNPDGSD DVTDEEGDGE VLEGQKGSPK KSAEPKVHAQ EEGNKDELKS KATKAKADAT EAVKAAELAK [ 120]

#Thai-T107 MKQLCGLAFL ALLLNFLTCD NVATRGEIVN LKNTNLRNGW SMKNVSAQNE ENIVHSDGSD DVTDKEEDGE VLEGQNGSPK KSAEQKVHAQ EEVNKESLKS KATTAKTEAE KAANAADLAK [ 120]

#Thai-113 MKQLCGLAFL ALLLNLLTCG NVATRGEIVN LKNTNLRNGW SMNNLSAQNE ENIVHSDGSD DATDKEEDGE VLEGQKGSPK KSAEQKVHAQ EEGNKDELKS KATTAKTEAE KAANAADLAK [ 120]

#Thai-430 MKQFCGLAFL ALLLNFLTCD NVATRGEIVN LKNPNLRNGW SMNNLSAQNE ENIVHSDGSD DVTDKEEDGE VLEGQKGSPK KSAEPKVHAQ EEGNKDELKS KATKAKADAT EAVKAAELAK [ 120]

#China-CG11 MKQLCGLAFL ALLLNFLTRD NVATRGEIVN LKNTNLRNGW SMKNLSAQNE ENIVNSHGSD DVTDKEENGE VLEGQNGSPK KLAEPKVHAQ EEVNKESLKS KATTAKTEAE KAANAADLAK [ 120]

#AY454083-Chess MKQLCGLAFL ALLLNFLTCD NVATRGEIVN LKNPNLRNGW SMKNVSAQNE ENIVNSHGSD DVTDKEENGE VLEGENGSPK KLAEPKVHAQ EEETKESLKS KAQNAKAEAE KAAKAAESAK [ 120]

#Thai-TC103 MKQLCGLAFL ALLLNFLTCD NVATRGEIVN LKNTNLRNGW SMNNLSAQNE ENIVNSHGSD DVTDKEEDGE VLEGQNGSPK KLAEPKVHAQ EEETKDELKS KATKAKTEAV EAAKAAETAK [ 120]

#Thai-TV400 MKQLCGLAFL ALLLNFLTCD NVATRGEIVN LKNPNLRNGW SMKNLSAQNE ENIVNPDGSD DVTDEEGDGE ALEGQKGSPK KSAEPKVHAQ EEGNKDELKS KATKAKADAT EAVKAAELAK [ 120]

#AY454089-Br781T ********** ********** ********** ********** SMNNLSAQNE ENIVNSHGSD DVTDKEEDGE VLEGQNGSPE KSAEPKVHAQ EEETKESLKS KAQNAKAEAE KAAKAAESAK [ 120]

#PVNG01493-NK MKQFCGLAFL ALLLNFLTCD NVATRGEIVN LKNTNLRNGW SMKNLSAQNE ENIVHSDGSD DVTDKEEDGE VLEGQNGSPK KLAEPKVHAQ EAESKDELKS KATKAKTEAV EAANAAELAK [ 120]

#Thai-411 MKQLCGLAFL ALLLNFLTRD NVATRGEIVN LKNPNLRNGW SMNNLSAQNE ENIVNPDGSD DVTDEEGDGE ALEGQNGSPE KSAEPKVHAQ EAETKESLKL KATKAKTEAV EAAKAAALAK [ 120]

#Thai-TF83 MKQLCGLAFL ALLLNFLTCD NVATRGEIVN LKNTNLRNGW SMNNLSAQNE ENIVNSDGSD DVTDKEENGE VLEGENGSPK KLAEPKVHAQ EAETKESLKL KATKAKTEAV EAAKAAASAK [ 120]

#Thai-109 MKQLCGLAFL ALLLNFLTCD NVATRGEIVN LKNTNLRNGW SMKNVSAQNE ENIVHSYGSD DVTDKEEDGE VLEGENGSPE KSAEPKVHAQ GAESKDELKS KATKAKTEAV EAANAAELAK [ 120]

#Thai-410 MKQLCGLAFL ALLLNFLTRD NVATRGEIVN LKNPNLRNGW SMNNLSAQNE ENIVNSHGSD DVTDKEENGE VLEGQKGSPK KSAEQKVHAQ EEETKESLKS KAQNAKAEAE KAAKAAESAK [ 120]

#AY454098-Thai ********** ********** ********** ********** SMKNVSAQNE ENIVHSDGSD DVTDKEEDGE VLEGENGSPE KSAEPKVHAQ EAESKDELKS KATKAKTEAV EAANAAELAK [ 120]

#AF099662-Belem MKQLCGLAFL ALLLNFLTCD NVATRGEIVN LKNPNLRNGW SMNNLSAQNE ENIVNPDGSD DVTDEEGDGE ALEGQNGSPE KSAEPKVHAQ EEVNKESLKS KAQNAKAEAE KAAKAAESAK [ 120]

#AY454080-Br1B MKQFCGLAFL ALLLNFLTCD NAATRGEIVN LKNPNLRNGW SMNNLSAQNE ENIVNPDGSD DVTDEEGDGE ALEGQNGSPE KSAEPKVHAQ EEVNKESLKS KAQNAKAEAE KAAKAAESAK [ 120]

#AY454085-Br56 ********** ********** ********** ********** SMKNLSAQNE ENIVNSHGSD DVTDKKEDGE ALEGQNGSPK KSAEPKVHAQ EAETKESLKL KAANAKKDAE EAAKAAQSAK [ 120]

#Thai-115 MKQLCGLAFL ALLLNFLTCD NVATRGEIVN LKNPNLRNGW SMKNLSAQNE ENIVNSHGSD DVTDKKEDGE ALEGENGSPK KLAEPKVHAQ EAESKDELKS KATKAKTEAV EAANAAELAK [ 120]

#Thai-TF91 MKQLCGLAFL ALLLNFLTCD NVATRGEIVN LKNTNLRNGW SMKNLSAQNE ENIVHSDGSD DVTDKEEDGE VLEGENGSPK KLAEPKVHAQ EAENKESLKL KATKAKTEAV EAANAAELAK [ 120]

#Thai-126 MKQLCGLAFL ALLLNFLTCD NVATRGEIVN LKNPNLRNGW SMNNLSAQNE ENIVNSHGSD DVTDKEEDGE VLEGQNGSPE KSAEPKVHAQ EEVNKESLKS KAQNAKAEAE KAAKAAESAK [ 120]

#Thai-442 MKQLCGLAFL ALLLNFLTCD NVATRGEIVN LKNPNLRNGW SMKNLSAQNE ENIVNSHGSD DVTDKKEDGE ALEGENGSPK KLAEPKVHAQ EAESKDELKS KATKAKTEAV EAANAAELAK [ 120]

#Thai-408 MKQLCGLAFL ALLLNFLTRD NVATRGEIVN LKNTNLRNGW SMKNLSAQNE ENIVNPDGSD DVTDKKEDGE ALEGENGSPK KLAEPKVHAQ EAESKDELKS KATKAKTEVV EAANAAELAK [ 120]

#India-IN1 MKQLCGLAFL ALLLNFLTCD NVATRGEIVN LKNPNLRNGW SMKNLSAQNE ENIVNPDGSD DVTDEEGDGE ALEGQKGSPK KSAEPKVHAQ EEGNKDELKS KATKAKADAT EAVKAAELAK [ 120]

#Thai-TB123 MKQLCGLAFL ALLLNFLTRD NVATRGEIVN LKNTNLRNGW SMKNLSAQNE ENIVNSHGSD DVTDKKEDGE ALEGENESPK ESAEPKVHAQ EAENKDELKS KAATAKEEAE KAAKAAASAK [ 120]

#Thai-T139 MKQLCGLAFL ALLLNFLTRD NAATRGEIVN LKNPNLRNGW SMNNLSAQNE ENIVHSDGSD DVTDKEEDGE VLEGENGSPK KLAEPKVHAQ EAETKESLKL KATKAKTEAV EAANAAELAK [ 120]

#Thai-TF127 MKQLCGLAFL ALLLNFLTCD NVATRGEIVN LKNPNLRNGW SMKNLSAQNE ENIVNPDGSD DVTDEEGDGE ALEGQNGSPE KSAEPKVHAQ EAETKESLKL KATKAKTEAV EAAKAAALAK [ 120]

#Thai-104 MEQLCGLAFL ALLLNFLTRD NVATRGEIVN LKNTNLRNGW SMKNPSAQNE ENIVRSDGSD DVTDKEEDGE VLEGENGSPK KLAEPKVHAQ EAESKESLKL KAAHAKKDAE EAAKAAQSAR [ 120]

#Thai-413 MKQLCGLAFL ALLLNFLTRD NVATRGEIVN LKNTNLRNGW SMKNLSAQNE ENIVNSHGSD DVTDKKEDGE ALEGENESPK ESAEPKVHAQ EAENKDELKS KAATAKEEAE KAAKAAASAK [ 120]

#Thai-431 MKRLCGLAFL ALLLNFLTCD NVATRGEIVN LKNPNLRNGW SMKNVSAQNE ENIVHSDGSD DVTDKEEDGE VLEGENGSPK KLAEPKVHAQ EAESKESLKL KAANAKKDAE EATKAAQSAK [ 120]

#Thai-447 MKQLCGLAFL ALLLNFLTRD NVATRGEIVN LKNTNLRNGW SMKNLSAQNE ENIVHSEGGD DVTDKEEDGK VLEGENGSPK RLAEPKAHAQ EAESKESLKL KAANAKKDAE EAAKAAQSAK [ 120]

#Thai-465 MKQLCGLAFL ALLLNFLTCD NVATRGEIVN LKNPNLRNGW SMKNVSAQNE ENIVHSDGSD DVTDKEEDGE VLEGENGSPK KLAEPKVHAQ EAESKESLKL KAANARKDAE EAAKAAQSAK [ 120]

#Thai-425 MKQFCGLAFL ALLLNFLTCD NVATRGEVVN LKNPNLRNGW SMKNLSAQNE ENIVNPDGSD DVTDEEGDGE ALEGQNGSPE KSAEPKVHAQ EAETKESLKL KATKAKTEAV EAAKAAALAK [ 120]

#Thai-421 MKQFCGLAFL ALLLNFLTCD NVATRGEIVN LKNPNLRNGW SMKNLSAQNE ENIVNPDGSD DVTDEEGDGE VLEGQNGSPE KSAEPKVHAQ EEETKESLKS KAQNAKAEAV EAANAAELAK [ 120]

#Papau-PNN1 MKQLCGLAFL ALLLNFLTCD NVATRGEIVN LKNTNLRNGW SMKNLSAQNE ENIVNPDGSD DVTDEEGDGE ALEGQNGSPE KSAEPKVHAQ EAETKESLKL KATKAKTEAV EAAKAAALAK [ 120]

#China-CG15 MKQLCGLAFL ALLLNFLTCD NVATRGEIVN LKNTNLRNGW SMKNLSAQNE ENIVHSDGSD DVTDKEEDGE VLEGENGSPE KSAEPKVHAQ EAETKESLKS KAQNAKAEAE KAAKAAESAK [ 120]

#AY454087-Br67T ********** ********** ********** ********** SMNNLSAQNE ENIVNPDGSD DVTDEEGDGE ALEGQNGSPE KSAEPKVHAQ EAETKESLKS KAQNAKAEAE KAAKAAELAK [ 120]

#AY454088-Br781B ********** ********** ********** ********** SMKNLSAQNE ENIVNPDGSD DVTDKKEDGK ALEGQNGSPK KSAEPKVHAQ EAETKESLKL KAANAKKDAE EAAKAAQSAK [ 120]

#AY454090-EcuB ********** ********** ********** ********** SMNNLSAQNE ENIVNPDGSD DVTDEEGDGE ALEGQNGSPE KSAEPKVHAQ EEVNKESLKS KAQNAKAEAE KAAKAAESAK [ 120]

#AY454094-Ong ********** ********** ********** ********** SMKNVSAQNE ENIVNPDGSD DVTDEEGDGE ALEGQNGSPE KSAEPKVHAQ EAETKESLKL KATKAKTEAV EAAKAAALAK [ 120]

#AY454086-Br67B ********** ********** ********** ********** SMNNLSAQNE ENIVNPDGSD DVTDEEGDGE ALEGQNGSPE KSAEPKVHAQ EAETKESLKS KAQNAKAEAE KAAKAAELAK [ 120]

#Thai-105 MKQFCGLAFL ALLLNLLTCD KVATRGEIVN LRNTNLRNGW SMKNLSAQNE ENILHPDGSD DVTDKEEDGE VLEGQKGSPK KSAEQKVHAQ EEETKESLKS KAQNAKAEAE KAAKAAESAK [ 120]

#PVMG01384-Mauri MKQLCGLAFL ALLLNFLTCD NVATRGEIVN LKNPNLRNGW SMNNLSAQNE ENIVNSHGSD DVTDKEENGE VLEGENESPE KSAEPKVHAQ EEETKESLKS KAQNAKAEAE KAAKAAESAK [ 120]

#PVIIG04181-Ind MKQLCGLAFL ALLLNFLTCD NVATRGEIVN LKNPNLRNGW SMKNLSAQNE ENIVNSHGSD DVTDKEEDGE VLEGQNGSPK KSAEPKVHAQ EAETKESLKL KATKAKTEAV EAANAAELAK [ 120]

#PVBG05499-Brazi MKQFCGLAFL ALLLNFLTRD NAATRGEIVN LKNPYLRNGW SMKNLSAQNE ENIVHSDGSD DVTDKEEDGE VLEGENESPK KSAEPKVHAQ EAESKESLKL KAANAKKDAE EAAKAAQSAK [ 120]

Conserved >< Semi-conserved

#XM001613146-Sal ENTLDALEKV NVPTELNNEK NFAESAATEA KKQEKISTEA AE----EVKE IEVDGQLEKL KNEEE---KT AKKARKQEIK TEIAEQAAKA QAAK------ -TEAETAQK- ------DATT [ 240]

#AY454081-Br1T KNTLDALEKV NVPTELNNVK KFAESAATEA QKQENIATEA EKK---VAEA NGEVVELQKL KDEVD---KA AKKAKQLQLK AEIAEHAVKA QVAK------ -TEAEKAQK- ------DATT [ 240]

#AY454082-Br69 KNTLDALEKV NVPTELNNVK KFAESAATEA QKQENIATEA EKKV---AEA NGEVVELQKL KDEVKKAEKA AKKAKKLQIK AQIAEQAIKA QVAK------ -TEAKKAQKD AEKAKTEATT [ 240]

#AY454092-India DRTLVALEKV DVPTELDKVK EFAVSAATQA KNQETIATKA AES----AEA IGGDGDLGNL KSEVENAEKA AKKAKQLQIK AEIAEQAVMA QAAK------ -TEAKKAQKD AEKAKTEATT [ 240]

#AY454091-EcuT DRTLVALEKV KVPTELNKAK EFAEAAAAEA KNQENLA-IA AEK---AAQE MEVNEDLQNL KTEVEKAEKA AKKAKQLEIK VQIAEQAAKA QVAK------ -TEAEKAQT- ------DATA [ 240]

#Thai-414 DSTLNALEKV KVPTELNKAK EFAEAAAAEA KNQENLA-IA AEK---AAQE MEVNGDLQNL KTEVEKAEKA AKKAKQLEIK VQIAEQAAKA QVAK------ -TEAEKAQK- ------DATA [ 240]

#Thai-129 DGTLNALEKV KVPTELNKAK EFAEAAAAEA KKQENIA-IA AEK---AAQE MEVNEDLQNL KTEVEKAEKA AKKAKQLEIK VQKAEQAAKA QVAK------ -TEAEKAQT- ------DATA [ 240]

#Thai-T120 DSTLNALEKV KVPTELNKAK EFAEAAAAEA KKQENLA-IA AEK---AAQE MEVNEDLQNL KTEVEKAEKA AKKAKQLEIK VQIAEQAAKA QVAK------ -TEAEKAQK- ------DATA [ 240]

#Thai-462 DSTLNALEKV KVPTELNKAK EFAEAAAAEA KNQENLA-IA AEK---AAQE MEVNEDLQNL KTEVEKAEKA AKKAKQLEIK VQIAEQAAKA QVAK------ -TEAEKAQT- ------DATA [ 240]

#China-CG12 DRTLVALEKV KVPTELNKAK EFAEAAAAEA KNQENLA-IA AEK---AAQE MEVNEDLQNL KTEVEKAEKA AKKAKQLEIK VQIAEQAAKA QVAK------ -TEAEKAQK- ------DATA [ 240]

#China-CG8 NNTLDALKKV KVPTELNKAK EFAESAATEA KKQENIATEA EK---KVAEA NGEVVELQKL KDEVKKAEKA AKKAKKLQIK AQIAEQAIKA QVAK------ -TEAETAQT- ------EATA [ 240]

#AY454084-Bangl KNTLDALAKV KVPTELSKAN EFAESAVTEA KKQEEIA--- -------AKE IEVDGEFQKL KDEVDKAEKA AKKAKKLQIK VQIVEQAAKA QVAK------ -TEAETAQK- ------EATA [ 240]

#AY454097-SL57 ENILVALKKV DVPTELDKVK EFAVSAATQA KNQETIATKA AES----AEA IGDDGDLGNL KSEVENAEKA AKKAKQLQIK AEIAEQAVMA QAAK------ -TEAKKAQKD AEKAKTEATT [ 240]

#AY454096-SL ENILVALKKV DVPTELDKVK EFAVSAATQA KNQETIATKA AES----AEA IGDDGDLGNL KSEVENAEKA AKKAKQLQIK AEIAEQAVMA QAAK------ -TEAKKAQKD AEKAKTEATT [ 240]

#Thai-106 KNTWVALEKV NVTTELDKVK EFAESAVTEA KKQEQIATEV ETKV---AKA TGEDGELQKL KEEVEKAGRA AKKAKKLQIR VQIAEQAANA QVAK------ -TEAETAQK- ------DATA [ 240]

#Thai-NR1 KNTLDALAKV KVPTELSKAN EFAESAVTEA KKQEEIA--- -------AKE IEVDGEFQKL KDEVDKAEKA AKKAKKLQIK VQIVEQAAKA QVAK------ -TEAETAQK- ------EATA [ 240]

#Thai-T107 KNTWVALEKV NVTTELDKVK EFAESAVTEA KKQEQIATEV ETKV---AKA TGEDGELQKL KEEVEKAERA AKKAKKLQIK VQIAEQAANA QVAK------ -TEAETAQK- ------DATA [ 240]

#Thai-113 KNTWVALEKV NVSTELDKVK EFAESAVTEA KKQEQIATEV ETKV---AEA TGEDGELQKL KEEVEEAERA AKKAKKLQIK VQIAEQAANA QVAK------ -TEAETAQK- ------DATA [ 240]

#Thai-430 KNTLDALAKV KVPTELSKAN EFAESAVTEA KKQEEIA--- -------AKE IEVDGEFQKL KDEVDKAEKA AKKAKKLQTK VQIVEQAAKA QVAK------ -TEAETAQK- ------EATA [ 240]

#China-CG11 KNTWVALEKV NVTTELDKVK EFAESAVTEA KKQEQIATEV ETKV---AKA TGEDGELQKL KEEVEKAERA AKKAKKLQIK VQIAEQAANA QVAK------ -TEAETAQK- ------DATA [ 240]

#AY454083-Chess DSTLDALKKV KVPTEHDKVK KFAESAATEA KKQENLAIEA EK----AAQA IEDDGQKEKL KTEVDKAEKA AKKAKQLQIK AEIAEQAAKA QLAKTEAEKA KTEAETAQK- ------DATA [ 240]

#Thai-TC103 ENILVALKKV NESTELNKAK EFAESAATEA KKQETIATEA ETK---VSEG NGDDGELGNL KTEVDKAEKA AKKAKQLQIK AQIAEQAVKA QVAK------ -TEAETAQK- ------DATA [ 240]

#Thai-TV400 DRTLDALKKV KVPTEHDKVK KFAESAATEA KKQENLAIEA EK----AAQA IEDDGQKEKL KTEVDKAEKA AKKAKQLQIK AEIAEQAAKA QLAKTEAEKA KTEAETAQK- ------DATA [ 240]

#AY454089-Br781T ENTLDALEKV NVPTELNNVK KFAESAATEA KKQEKISTEA AE----EVKE IEVDGQLEKL KNEEE---KT AKKARKQEIK TEIAEQAAKA QAAK------ -TEAETAQK- ------DATT [ 240]

#PVNG01493-NK NNTLVALAKV KVPTEHDKVK KFAESAATEA KKQEKISTEA AE----EVKE IEVDGQLEKL KNEEE---KT AKKARKQEIK TEIAEQAAKA QAAK------ -TEAETAQK- ------DATT [ 240]

#Thai-411 DNTLVALKKV KVPTEHDKVK KFAELAATEA KKQEEIAIAA EKEAAKEAEA IEVDGEFKKL KDEVKKAEKA AKKAKKLQIK AQIAEQAIKA QVAK------ -TEAETAQT- ------EATA [ 240]

#Thai-TF83 DRTLVALEKV NVPTELNNVK KFAESAATEA KNQETIATKA AE----SAEA IGDDGDLGNL KSEVENAEKA AKKARKQEIK TEIAEQAAKA QAAK------ -TEAETAQK- ------DATT [ 240]

#Thai-109 NNTLVALAKV KVPTGHDKVK KFAESAATEA KKQEKISTEA AE----EVKE IEVDGQLEKL KNEEK---KT AKKARKQEIK TEIAEQAAKA QAAK------ -TEAETAQK- ------DATT [ 240]

#Thai-410 KNTLDALEKV NVPTELNNVK KFAESAATEA QKQETIATKA AE----SAEA IGDDGDLGNL KSEVENAEKA AKKARKQEIK TEIAEQAAKA QAAK------ -TEAETAQK- ------DATT [ 240]

#AY454098-Thai NNTLVALAKV KVPTEHDKVK KFAESAATEA KKQEKISTEA AE----EVKE IEVDGQLEKL KNEEE---KT AKKARKQEIK TEIAEQAAKA QAAK------ -TEAETAQK- ------DATT [ 240]

#AF099662-Belem KNTLDALEKV NVPTELNNVK KFAESAATEA QKQENIATEA EKK---VAEA NGEVVELQKL KDEVD---KA AKKAKQLQLK AEIAEHAVKA QVAK------ -TEAEKAQK- ------DATT [ 240]

#AY454080-Br1B KNTLDALEKV NVPTELNNVK KFAESAATEA QKQENIATEA EKK---VAEA NGEVVELQKL KDEVD---KA AKKAKQLQLK AEIAEHAVKA QVAK------ -TEAEKAQK- ------DATT [ 240]

#AY454085-Br56 NSTLDALKKV KVPTELNKAK EFAESAATEA KKQETIATEA EKK---VAEA NGEVVELQKL KDEV---DKA AKKAKQLQLK AEIAEHAVKA QVAK------ -TEAEKAQK- ------DATT [ 240]

#Thai-115 NNTLDALKKV KVPTELNKAK EFAESAATEA KKQENIATEA EKK---VAEA NGEVVELQKL KDEVKKAEKA AKKAKQLEIK VQIAEQAAKA QVAK------ -TEAEKAQT- ------DATA [ 240]

#Thai-TF91 NNTLDALKKV KVPTELNKAK EFAESAATEA KKQENIATEA EKK---VAEA NGEVVELQKL KDEVD---KA AKKAKQLQLK AEIAEHAVKA QVAK------ -TEAEKAQK- ------DATT [ 240]

#Thai-126 KNTLDALEKV NVPTELNNVK KFAESAATEA QKQENIATEA EKK---VAEA NGEVVELQKL KDEVD---KA AKKAKQLQLK AEIAEHAVKA QVAK------ -TEAEKAQK- ------DATT [ 240]

#Thai-442 NNTLDALKKV KVPTELNKAK EFAESAATVA KKQENIATEA EKK---VAEA NGEVVELQKL KDEVKKAEKA AKKAKQLEIK VQIAEQAAKA QVAK------ -TEAEKAQT- ------DATA [ 240]

#Thai-408 NNTLDALKKV KVPTELNKAK EFAEAAAAEA KKQENIATEA EKK---VAEA NGEVVELQKL KDEVKKAEKA AKKAKQLEIK VQIAEQAAKA QVAK------ -TEAEKAQT- ------DATA [ 240]

#India-IN1 DRTLVALEKV KVPTELNKAK EFAEAAAAEA KKQENLAIAA EK----AAQE MEVNEDLQNL KTEVEKAEKA AKKAKQLEIK VQIAEHAVKA QVAK------ -TEAEKAQK- ------DATT [ 240]

#Thai-TB123 DRTLVALEKV KVPTELNKAK EFAEAAAAEA KNQENLA-IA AEK---AAQE MEVNEDLQNL KTEVEKAEKA AKKAKQLEIK VQIAEQAAKA QVAK------ -TEAEKAQT- ------DATA [ 240]

#Thai-T139 NNTLDALKKV KVPTELNKAK EFAESAATEA KKQENIATEA EKK---VAEA NGEVVELQKL KDEVKKAEKA AKKAKQLQLK AEIAEHAVKA QVAK------ -TEAEKAQK- ------DATT [ 240]

#Thai-TF127 DNTLVALKKV KVPTELNKAK EFAESAATEA KKQENIATEA EKK---VAEA NGEVVELQKL KDEV---DKA AKKAKQLQLK AEIAEHAVKA QVAK------ -TEAEKAQK- ------DATT [ 240]

#Thai-104 DSTLNALEKV KVPTELNKAK EFAEAAAAEA KNQENLATAA AEK---AAQE MEVNEDLQNL KTEVEKAEKA AKKAKQLEIK VQIAEQAAKA QVAK------ -TEAEKAQT- ------DATA [ 240]

#Thai-413 DRTLVALEKV KVPTELNKAK EFAEAAAAEA KNQENLA-IA AEK---AAQE MEVNEDLQNL KTEVEKAEKA AKKAKQLEIK VQIAEQAAKA QVAK------ -TEAEKAQT- ------DATA [ 240]

#Thai-431 DSTLNALEKV KVPTELNKAK EFAEAAAAEA KNQENLA-IA AEK---AAQE MEVNEDLQNL KTEVEKAEKA AKKAKQLEIK VQIAEQAAKA QVAK------ -TEAEKAQK- ------DATA [ 240]

#Thai-447 DSTLNALEKV KVPTEFYRAK ESAEAATAEA ENQENLA-IA AEK---AAQE MEVNEDLQNL KTEVEKAEKA AKKAKQLEIK VQIAEQAAKA QVAK------ -TEAKKAQT- ------DATA [ 240]

#Thai-465 DSTLNALEKV KVPTELNKAK EFAEAAAAEA KNQENLA-IA AEK---AAQE MEVNEDLQNL KTEVEKAEKA AKKAKQLEIK VQIAEQAAKA QVAK------ -TEAKKAQT- ------DATA [ 240]

#Thai-425 DNTLVALKKV KVPTELNKAK EFAESAATEA KKQENIATEA EKK---VAEA NGEVVELQKL KDEV---NKA AKKAKQLQLK AEIAEHAVKA QVAK------ -TEAEKAQK- ------DATT [ 240]

#Thai-421 NNTLDALKKV KVPTELNKAK EFAESAATEA KKQENIATEA EK---KVAEA NGEVVELQKL KDEVKKAEKA AKKAKKLQIK AQIAEQAIKA QVAK------ -TEAETAQT- ------EATA [ 240]

#Papau-PNN1 DNTLVALKKV KVPTEHDKVK KFAELAATEA KKQEEIAIAA EKEAAKEAEA IEVDGEFKKL KDEVKKAEKA AKKAKKLQIK AQIAEQAIKA QVAK------ -TEAETAQT- ------EATA [ 240]

#China-CG15 KNTLDALEKV NVPTELNNVK KFAESAATEA KKQETIATEA ETK---VSEG NGDDGELGNL KTEVDKAEKA AKKAKQLQLK AEIAEHAVKA QVAK------ -TEAEKAQK- ------DATT [ 240]

#AY454087-Br67T NNTLDALKKV KVPTELNKAK EFAESAATEA QKQENIATEA EKK---VAEA NGEVVELQKL KDEV---DKA AKKAKQLQLK AEIAEHAVKA QVAK------ -TEAEKAQK- ------DATT [ 240]

#AY454088-Br781B DSTLNALEKV KVPTELNKAK EFAEAAAAEA KNQENLA-IA AEK---AAQE MEVNEDLQNL KTEVEKAEKA AKKAKQLEIK VQIAEQAAKA QVAK------ -TEAEKAQT- ------DATA [ 240]

#AY454090-EcuB KNTLDALEKV NVPTELNNVK KFAESAATEA QKQENIATEA EKK---VAEA NGEVVELQKL KDEVD---KA AKKAKQLQLK AEIAEHAVKA QVAK------ -TEAEKAQK- ------DATT [ 240]

#AY454094-Ong DNTLVALKKV KVPTEHDKVK KFAELAATEA KKQEEIAIAA EKEAAKEAEA IEVDGEFKKL KDEVKKAEKA AKKAKKLQIK AQIAEQAIKA QVAK------ -TEAETAQT- ------EATA [ 240]

#AY454086-Br67B NNTLDALKKV KVPTELNKAK EFAESAATEA QKQENIATEA EKK---VAEA NGEVVELQKL KDEV---DKA AKKAKQLQLK AEIAEHAVKA QVAK------ -TEAEKAQK- ------DATT [ 240]

#Thai-105 KNTLDALEKV NVPTELNNVK KFAESAATEA QKQETIATKA AES----AEA IGDDGDLGNL KSEVENAEKA AKKAKQLQIK AEVAEQAVMA QAAK------ -TEAKKAQK- ------DAEK [ 240]

#PVMG01384-Mauri KNTLDALEKV NVPTELNNVK KFAESAATEA KNQETIATKA AES-----EA IGDDGDLGNL KTEVDKAEKA AKKAKQLQIK AQIAEQAVKA QVAK------ -TEAETAQK- -------ATT [ 240]

#PVIIG04181-Ind NRTLDALKKV KVPTELNKAK EFAESAATEA KKQENIATEA EKK----AEA NGEVVELQKL KDEVKKAEKA AKKAKQLQLK AEIAEHAVKA QVAK------ -TEAEKAQK- -------ATT [ 240]

#PVBG05499-Brazi DSTLNALEKV KVPTELNKAK EFAEAAAAEA KNQENLAIAA EK-----AQE MEVNEDLQNL KTEVEKAEKA AKKAKQLEIK VQIAEQAAKA QVAK------ -TEAEKAQT- -------ATA [ 240]

#XM001613146-Sal AK-------D EAIKETGKPK SQ-------- --NTTKAVTM ATEEEKKTKD EAQTASEKAG KTAEEAQKEV GKETADDDKE VSQLEEEIKE LERILKIVKD LASEASSASD NAKKAKLKTQ [ 360]

#AY454081-Br1T AK-------E VAIKETGTSK SE-------- --NVTKAIDM AKKEEEETKN QASIASENAD KAAKAAQEEV KKEIKDENKE ISQLENEITK LGDILNTVKE LASNAEDASK NAKKEMAKAQ [ 360]

#AY454082-Br69 AK-------E ETIKETGTSE SE-------- --IVTKAVAT ATAEEEKTQK EAQTASEKAD KAVEETQKEV DKGIEDESKE TSDLE----- --DILKSVKE LASSAEDASK NAKKEMTKAQ [ 360]

#AY454092-India AK-------E ETIKETGTSE SE-------- --IVTKAVAT ATAEEEKTQK EAQTASEKAD KAVEETQKEV DKGIEDESKE TSDLE----- --DILKSVKE LASSAEDASK NAKKEMAKAQ [ 360]

#AY454091-EcuT AK-------E VAIKETDKLN SQ-------- --NATKAVTT ATEQEEETKK EAKTASENAD KAAKGAQEEV EKEIKDESKE KLELE----- --GILNTLKT LASNAEDASK KAQKAKLKAQ [ 360]

#Thai-414 AK-------E VAIKETDKLN SQ-------- --NATKAVTT ATEQEEETKK EAKTASENAD KAAKGAQEEV EKEIKDESKE KLELE----- --GILNTLKT LASNAEDASK KAQKAKLKAQ [ 360]

#Thai-129 AK-------E VAIKETDKLN SQ-------- --NATKAVTT ATEQEEETKK EAKTASENAD KAAKGAQEEV EKEIKDESKE KLELE----- --GILNTLKT LASNAEDASK KAQKAKLKAQ [ 360]

#Thai-T120 AK-------E VAIKETDKLN SQ-------- --NATKAVTT ATEQEEETKK EAQTAVKMQI SSKRSSREEV EKEIKDESKE KLELE----- --GILNTLKT LASNAEDASK KAQKAKLKAQ [ 360]

#Thai-462 AK-------E VAIKETDKLN SQ-------- --NATKAVTT ATEQEEETKK EAKTASENAD KAAKGAQEEV EKEIKDESKE KLELE----- --GILNTLKT LASNAEDASK KAQKAKLKAQ [ 360]

#China-CG12 AK-------E VAIKETDKLN SQ-------- --NATKAVTT ATEQEEETKK EAKTASENAD KAAKGAQEEV EKEIKDESKE KLELE----- --GILNTLKT LASNAEDASK KAQKAKLKAQ [ 360]

#China-CG8 AK-------E EAIKETDKPK LQ-------- --NATKAVTM TTEEEEKTKK EAKTASEKAG NAAEEAQKEE KKGIADEDKE ISDLEIEIKE LEGILNKVKD LASNAEVASK KAKKANLKTQ [ 360]

#AY454084-Bangl AK-------D EAIKETGTSK SE-------- --NATKAVAM ATEEEEKTKN QANIASENAD NAAKEAQKEV EKEITDEDKE ITELG----- --DILKKVEV LASDASSASE EAKKANLKIQ [ 360]

#AY454097-SL57 AK-------E ETIKETGTSE SE-------- --IVTKAVAT ATAEEEKTQK EAQTASEKAD KAVEETQKEV DKGIEDESKE TSDLE----- --DILKSVKE LASSAEDASK NAKKEMAKAQ [ 360]

#AY454096-SL AK-------E ETIKETGTSE SE-------- --IVTKAVAT ATAEEEKTQK EAQTASEKAD KAVEETQKEV DKGIEDESKE TSDLE----- --DILKSVKE LASSAEDASK NAKKEMAKAQ [ 360]

#Thai-106 AK-------E EAIKETDKPK LQ-------- --NETKAVAM VKKEEEETKK EAKTASEKAG NAAEKAQKEV KKGTADEGKR ISDLENEISE LEDILKEVGA LASQASSASE KAKTAKLKTQ [ 360]

#Thai-NR1 AK-------D EAIKETGTSK SE-------- --NATKAVAM ATEEEEKTKN QANIASENAD NAAKEAQKEV EKEITDEDKE ITELG----- --DILKKVEV LASDASSASE EAKKANLKIQ [ 360]

#Thai-T107 AK-------E EAIKETDKPK LQ-------- --NETKAVAM VKKEEEETKK EAKTASEKAG NAAEKAQKEV KKGTADEDKK ISDLENEISE LEDILKEVGA LASQASSASE KAKTAKLKTQ [ 360]

#Thai-113 AK-------E EAIKETDKPK LQ-------- --NETKAVAM VKKEEEETKK EAKTASEKAG NAAEKTQKEV KKGTADEDKK ISDLENEISE LEDILKEVGA LASQASSASE KAKTAKLKTQ [ 360]

#Thai-430 AE-------D EAIKETGTSK SE-------- --NATKAVAM ATEEEEKTKN QAIIASENAD NAAKEAQKEV EKEITDEDKE ITELG----- --DILKKVEV LASDASSASE EAKKANLKIQ [ 360]

#China-CG11 AK-------E EAIKETDKPK LQ-------- --NETKAVAM VKKEEEETKK EAKTASEKAG NAAQKAQKEV KKGTADEDKK ISDLENEISE LEDILKEVGA LASQASSASE KAKTAKLKTQ [ 360]

#AY454083-Chess AK-------E VALKETDTSK SQ-------- --YATKAVDM ATREEGKTKK EAQTASEKAD EAAKEAQKEV EKEIKDEDKD ISVLQNEITE LEGILETVKK LTSKASSALE EAKKAKLKTQ [ 360]

#Thai-TC103 AK-------E VALKETGTSK SQ-------- --YATKAVDM ATKEEGKTKN EAQTASENAD KAAKAAQEEV EKEIKDENKE ILELDNEITK LGGILSTVKP LVSEALSASE KAKKANLKTQ [ 360]

#Thai-TV400 AK-------E VALKETDTSK SQ-------- --YATKAVDM ATREEGKTKK EAQTASEKAD EAAKEAQKEV EKEIKDEDKD ISVLQNEITE LEGILETVKK LTSKASSALE EAKKAKLKTQ [ 360]

#AY454089-Br781T AK-------D EAIKETGKPK SQ-------- --NTTKAVTM ATEEEKKTKD EAQTASEKAG KTAEEAQKEV GKETADDDKE VSQLEEEIKE LERILKIVKD LASEASSASD NAKKAKLKTQ [ 360]

#PVNG01493-NK AK-------D EAIKETGKPK SQ-------- --NTTKAVTM ATEEEKKTKD EAQTASEKAG KTAEEAQKEV GKETADDDKE VSQLEEEIKE LERILKIVKD LASEASSASD NAKKAKLKTQ [ 360]

#Thai-411 AK-------E EAIKETDKPK LQ-------- --NATKAVTM TTEEGEKTKK EAKTASEKAG NAAEEAQKEE KKGIADEDKE ISDLEIEIKE LEGILNKVKD LASNAEVASK KAKKANLKTQ [ 360]

#Thai-TF83 AK-------D EAIKETGKPK SQ-------- --NTTKAVTM ATEEEKKTKD EAQTASEKAG KTAEEAQKEV GKETADDDKE VSQLEEEIKE LERILKIVKD LASEASSASD NAKKAKLKTQ [ 360]

#Thai-109 AK-------D EAIKETGKPK SQ-------- --NTTKAVTM ATEEEKKTKD EAQTAGEKAG KTAEEAQKEV GKETADDDKE VSHLEEEIKE LERILKIVKD LASEASSASD NAKKAKLKTQ [ 360]

#Thai-410 AK-------D EAIKETGKPK SQ-------- --NTTKAVTM ATEEEKKTKD EAQTASEKAG KTAEEAQKEV GKETADDDKE VSQLEEEIKE LERILKIVKD LASEASSASD NAKKAKLKTQ [ 360]

#AY454098-Thai AK-------D EAIKETGKPK SQ-------- --NTTKAVTM ATEEEKKTKD EAQTASEKAG KTAEEAQKEV GKETADDDKE VSQLEEEIKE LERILKIVKD LASEASSASD NAKKAKLKTQ [ 360]

#AF099662-Belem AK-------E VAIKETGTSK SE-------- --NVTKAIDM AKKEEEETKN QASIASENAD KAAKAAQEEV KKEIKDENKE ISQLENEITK LGDILNTVKE LASNAEDASK NAKKEMVKAQ [ 360]

#AY454080-Br1B AK-------E VAIKETGTSK SE-------- --NVTKAIDM AKKEEEETKN QASIASENAD KAAKAAQEEV KKEIKDENKE ISQLENEITK LGDILNTVKE LASNAEDASK NAKKEMVKAQ [ 360]

#AY454085-Br56 AK-------E VAIKETGTSK SE-------- --NVTKAIDM AKKEEEETKN QASIASENAD KAAKAAQEEV KKEIKDENKE ISQLENEIKE LEGILNKVKD LASNAEVASK KAKKANLKAQ [ 360]

#Thai-115 AK-------E VAIKETDKLN SQ-------- --NATKAVTT ATEQEEETKK EAKTASENAD KAAKAAQEEV KKEIKDENKE ISQLENEITK LGDILNTVKE LASNAEDASK KAQKAKLKAL [ 360]

#Thai-TF91 AK-------E VAIKETGTSK SE-------- --NVTKAIDM AKKEEEETKN QASIASENAD KAAKAAQEEV KKEIKDENKE ISQLENEITK LGDILNTVKE LASNAEDASK NAKKEMVKAQ [ 360]

#Thai-126 AK-------E VAIKEAGTSK SE-------- --NVTKAIDM AKKEEEETKN QASIASENAD KAAKAAQEEV KKEIKDENEE ISQLEYEITK LGDILNTLKT LASNAEDASR KAQKAKLKAQ [ 360]

#Thai-442 AK-------E VAIKETDKLN SQ-------- --NATKAVTT ATEQEEETKK EGKTASENAD KAAKAAQEEV KKEIKDENKE ISQLENEITK LGDILNTVKE LASNAEDASK KAQKAKLKAQ [ 360]

#Thai-408 AK-------E VAIKETDKLN SQ-------- --NATKAVTT ATEQEEETKK EAKTASENAD KAAKAAQEEV KKEIKDENKE ISQLENEITK LGDILNTVKE LASNAEDASK NAKKEMVKAQ [ 360]

#India-IN1 AK-------E VAIKETGTSK SE-------- --NVTKAIDM AKKEEEETKN QASIASENAD KAAKAAQEEV KKEIKDENKE ISQLENEITK LGDILNTVKE LASNAEDASK NAKKEMVKAQ [ 360]

#Thai-TB123 AK-------E VAIKETDKLN SQ-------- --NATKAVTT ATEQEEETKK EAKTASENAD KAAKGAQEEV EKEIKDESKE KLELEG---- ---ILNTLKT LASNAEDASK KAQKAKLKAQ [ 360]

#Thai-T139 AK-------E VAIKETGTSK SE-------- --NVTKAIDM AKKEEEETKN QASIASENAG NAAEEAQKEE KKGIADEDKE ISDLEIEIKE LEGILNKVKD LASNAEVASK KAKKANLKTQ [ 360]

#Thai-TF127 AK-------E VAIKETGTSK SE-------- --NVTKAIDM AKKEEEETKN QASIASENAD KAAKAAQEEV KKEIKDENKE ISQLENEITK LGDILNTLKT LASNAEDASK KAQKAKLKAQ [ 360]

#Thai-104 AK-------E VAIKETDKLN SQ-------- --NATKAVTT ATEQEEETKK EAKTASENAD KAAKGAQEEV EKEIKDESKE KLELEG---- ---ILNTLKT LASNAEDASK KAQKAKLKAQ [ 360]

#Thai-413 AK-------E VAIKETDKLN SQ-------- --NATKAVTT ATEQEEETKK EAKTASGNAD KAAKGAQEVV EKEIKDESKE KLELEG---- ---ILNTLKT LASNAEDASK KAQKAKLKAQ [ 360]

#Thai-431 AK-------E VAIKETDKLN SQ-------- --NATKAVTT ATEQEEETKK EAKTASENAD KAAKGAQEEV EKEFKDESKE KLELEG---- ---ILNTLKT LASNAEDASK KAQKAKLKAQ [ 360]

#Thai-447 AK-------E VAIKETDNLN SQ-------- --NATKAVIT ATEQEEETKK EAKTASENAD KAVKGAQEEV EKEIKDESKE KLELEG---- ---ILNTLKT LASNAEDASK KAQKAKLKAQ [ 360]

#Thai-465 AK-------E VAIKETDKLN SQ-------- --NATKAVTT ATEQEEETKK EAKTASENAD KAVKGAQEEV EKEIKDESKE KLELEG---- ---ILNTLKT LASNAEDASK KAQKAKLKAQ [ 360]

#Thai-425 AK-------E VAIKETGTSK SENVTKAIDM AENVTKAIDM AKKEEEETKN QASIASENAD KAAKAAQEEV KKEIKDENKE ISQLENEITK LGDILNTLKT LASNAEDASK KAQKAKLKAQ [ 360]

#Thai-421 AK-------E EAIKETDKPK LQ-------- --NATKAVTM TTEEEEKTKK EAKTASEKAG NAAEEAQKEE KKGIADEDKE ISDLEIEIKE LEGILNKVKD LASNAEVASK KAKKANLKTQ [ 360]

#Papau-PNN1 AK-------E EAIKETDKPK LQ-------- --NATKAVTM TTEEEEKTKK EAKTASEKAG NAAEEAQKEE KKGIADEDKE ISDLEIEIKE LEGILNKVKD LASNAEVASK KAKKANLKTQ [ 360]

#China-CG15 AK-------E VAIKETGTSK SE-------- --NVTKAIDM AKKEEEETKN QASIASENAD KAAKAAQEEV KKEIKDENKE ISQLENEITK LGDILNTVKE LASNAEDASK NAKKEMVKAQ [ 360]

#AY454087-Br67T AK-------E VAIKETGTSK SE-------- --NVTKAIDM AKKEEEETKN QASIASENAD KAAKAAQEEV KKEIKDENKE ISQLENEIKE LEGILNKVKD LASNAEVASK KAKKANLKTQ [ 360]

#AY454088-Br781B AK-------E VAIKETDKLN SQ-------- --NATKAVTT ATEQEEETKK EAKTASENAD KAAKGAQEEV EKEIKDESKE KLELEG---- ---ILNTLKT LASNAEDASK KAQKAKLKAQ [ 360]

#AY454090-EcuB AK-------E VAIKETGTSK SE-------- --NVTKAIDM AKKEEEETKN QASIASENAD KAAKAAQEEV KKEIKDENKE ISQLENEITK LGDILNTVKE LASNAEDASK NAKKEMVKAQ [ 360]

#AY454094-Ong AK-------E EAIKETDKPK LQ-------- --NATKAVTM TTEEEEKTKK EAKIASEKAG NAAEEAQKEE KKGIADEDKE ISDLEIEIKE LEGILNKVKD LASNAEVASK KAKKANLKTQ [ 360]

#AY454086-Br67B AK-------E VAIKETGTSK SE-------- --NVTKAIDM AKKEEEETKN QASIASENAD KAAKAAQEEV KKEIKDENKE ISQLENEIKE LEGILNKVKD LASSAEVASK KAKKANLKTQ [ 360]

#Thai-105 AKSRSNYSEE ETIKETGTSE SE-------- --IVTKAVAT ATAEEEKTQK EAQTASEKAD KAVEETQKEV DKGIEDESKE TSDLED---- ---ILKSVKE LASSAEDASM NAKKEMAKAE [ 360]

#PVMG01384-Mauri AK-------D EAIKETGKPK SQ-------- ---TTKAVTM ATEEEKKTKD EAQTASEKAG KTAEEAQKEV GKETADDDKE VSQLEEEIKE LERILKIVKD LASEASSASD NAKKAKLKTQ [ 360]

#PVIIG04181-Indi AK-------E VAIKETGTSK SE-------- ---VTKAIDM AKKEEEETKN QASIASENAD KAAKAAQEEV KKEIKDENKE ISQLENEITK LGDILNTVKE LASNAEDASK NAKKEMVKAQ [ 360]

#PVBG05499-Brazi AK-------E VAIKETDKLN SQ-------- ---ATKAVTT ATEQEEETKK EAKTASENAD KAAKGAQEEV EKEIKDESKE KLELEG---- ---ILNTLKT LASNAEDASK KAQKAKLKAQ [ 360]

Semi-conserved >< Insert A

#XM001613146-Sal IAAEVVKAEK ARIEAEEAEK EAGEAKTKTE ATEKEVLKIS DESKAAKVKK AVEKAKEAEK QAKSEAEKAK GMADDAGGKG TTNLEDVLTK LSEVLTSVKS LASNAEVASK NAKKEMTKAQ [ 480]

#AY454081-Br1T IAAEVAKAEK AKIE------ ---------- ---------- ---------- ---------- ---------- ---------- ---------- ---------- ---------- ---------- [ 480]

#AY454082-Br69 IAAEVAKAEK AKIE------ ---------- ---------- ---------- ---------- ---------- ---------- ---------- ---------- ---------- ---------- [ 480]

#AY454092-India IAAEVAKAEK AKIE------ ---------- ---------- ---------- ---------- ---------- ---------- ---------- ---------- ---------- ---------- [ 480]

#AY454091-EcuT IAAEVAKAEK AKLE------ ---------- ---------- ---------- ---------- ---------- ---------- ---------- ---------- ---------- ---------- [ 480]

#Thai-414 IAAEVAKAEK AKLE------ ---------- ---------- ---------- ---------- ---------- ---------- ---------- ---------- ---------- ---------- [ 480]

#Thai-129 IAAEVAKAEK AKLE------ ---------- ---------- ---------- ---------- ---------- ---------- ---------- ---------- ---------- ---------- [ 480]

#Thai-T120 IAAEVAKAEK AKLE------ ---------- ---------- ---------- ---------- ---------- ---------- ---------- ---------- ---------- ---------- [ 480]

#Thai-462 IAAEVAKAEK AKLE------ ---------- ---------- ---------- ---------- ---------- ---------- ---------- ---------- ---------- ---------- [ 480]

#China-CG12 IAAEVAKAEK AKLE------ ---------- ---------- ---------- ---------- ---------- ---------- ---------- ---------- ---------- ---------- [ 480]

#China-CG8 IAAEVAKAEK AKIE------ ---------- ---------- ---------- ---------- ---------- ---------- ---------- ---------- ---------- ---------- [ 480]

#AY454084-Bangl IAAEVVKAEK AKLE------ ---------- ---------- ---------- ---------- ---------- ---------- ---------- ---------- ---------- ---------- [ 480]

#AY454097-SL57 IAAEVAKAEK AKIE------ ---------- ---------- ---------- ---------- ---------- ---------- ---------- ---------- ---------- ---------- [ 480]

#AY454096-SL IAAEVAKAEK AKIE------ ---------- ---------- ---------- ---------- ---------- ---------- ---------- ---------- ---------- ---------- [ 480]

#Thai-106 IAAEVVKAEK AKLE------ ---------- ---------- ---------- ---------- ---------- ---------- ---------- ---------- ---------- ---------- [ 480]

#Thai-NR1 IAAEVVKAEK AKLE------ ---------- ---------- ---------- ---------- ---------- ---------- ---------- ---------- ---------- ---------- [ 480]

#Thai-T107 IAAEVVKAEK AKLE------ ---------- ---------- ---------- ---------- ---------- ---------- ---------- ---------- ---------- ---------- [ 480]

#Thai-113 IAAEVVKAEK AKLE------ ---------- ---------- ---------- ---------- ---------- ---------- ---------- ---------- ---------- ---------- [ 480]

#Thai-430 IAAEVVKAEK AKLE------ ---------- ---------- ---------- ---------- ---------- ---------- ---------- ---------- ---------- ---------- [ 480]

#China-CG11 IAAEVVKAEK AKLE------ ---------- ---------- ---------- ---------- ---------- ---------- ---------- ---------- ---------- ---------- [ 480]

#AY454083-Chess IAAEVLKAEK ARIEAEEAEK EAGEAKTKTE TTQAEVLKIS NESKAAQVKK AVEKAKEAET QATSQAGNAK IKANDAGGKV TEDLEKETSD LEDILNTVKE LASNAEDASK NAKKEMVKAQ [ 480]

#Thai-TC103 IAAEVLKAEK ARIEAEEAEK EAGEAKKKTE ETGAEVLKIS DKPKATKVTQ EVEKAKAAEQ QAKTEAGKAK EKADAAGEKG TTKLENDLAE LNKILTSVES LASNAEDASK NAKKEMTKAQ [ 480]

#Thai-TV400 IAAEVLKAEK ARIEAEEAEK EAGEAKTKTE TTQAEVLKIS NESKAAQVKK AVEKAKEAET QATSQAGNAK IKANDAGGKV TEDLEKETSD LEDILNTVKE LASNAEDASK NAKKEMVKAQ [ 480]

#AY454089-Br781T IAAEVVKAEK ARIEAEEAEK EAGEAKTKTE ATEKEVLKIS DESKAAKVKK AVEKAKEAEK QAKSEAEKAK GMADDAGGKG TTNLEDVLTK LSEVLTSVKS LASNAEVASK NAKKEMTKAQ [ 480]

#PVNG01493-NK IAAEVVKAEK ARIEAEEAEK EAGEAKTKTE ATEKEVLKIS DESKAAKVKK AVEKAKEAEK QAKSEAEKAK GMADDAGGKG TTNLEDVLTK LSEVLTSVKS LASNAEVASK NAKKEMTKAQ [ 480]

#Thai-411 IAAEVAKAGK AKIE------ ---------- ---------- ---------- ---------- ---------- ---------- ---------- ---------- ---------- ---------- [ 480]

#Thai-TF83 IAAEVVKAEK ARIEAEEAEK EAGEAKTKTE ATEKEVLKIS DESKAAKVKK AVEKAKEAEK QAKSEAEKAK GMADDAGGKG TTNLEDVLTK LSEVLTSVKS LASNAEVASK NAKKEMTKAQ [ 480]

#Thai-109 IAAEVVKAEK ARIEAEEAEK EAGEAKTKTE ATEKEVLKIS DESKAAKVKK AVEKAKEAEK QAKSVAEKAK GMADDAGGKG TTNLEDVLTK LSEVLTSVKS LASNAEVASK NAKKEMTKAQ [ 480]

#Thai-410 IAAEVVKAEK ARIEAEEAEK EAGEAKTKTE ATEKEVLKIS DESKAAKVKK AVEKAKEAEK QAKSEAEKAK GMADDAGGKG TTNLEDVLTK LSEVLTSVKS LASNAEVASK NAKKEMTKAQ [ 480]

#AY454098-Thai IAAEVVKAEK ARIEAEEAEK EAGEAKTKTE ATEKEVLKIS DESKAAKVKK AVEKAKEAEK QAKSEAEKAK GMADDAGGKG TTNLEDVLTK LSEVLTSVKS LASNAEVASK NAKKEMTKAQ [ 480]

#AF099662-Belem IAAEVAKAEK AKIE------ ---------- ---------- ---------- ---------- ---------- ---------- ---------- ---------- ---------- ---------- [ 480]

#AY454080-Br1B IAAEVAKAEK AKIE------ ---------- ---------- ---------- ---------- ---------- ---------- ---------- ---------- ---------- ---------- [ 480]

#AY454085-Br56 IAAEVAKAEK AKIE------ ---------- ---------- ---------- ---------- ---------- ---------- ---------- ---------- ---------- ---------- [ 480]

#Thai-115 IAAEVAKAEK AKLE------ ---------- ---------- ---------- ---------- ---------- ---------- ---------- ---------- ---------- ---------- [ 480]

#Thai-TF91 IAAEVAKAEK AKIE------ ---------- ---------- ---------- ---------- ---------- ---------- ---------- ---------- ---------- ---------- [ 480]

#Thai-126 IAAEVAKAEK AKQE------ ---------- ---------- ---------- ---------- ---------- ---------- ---------- ---------- ---------- ---------- [ 480]

#Thai-442 IAAEVAKAEK AKLE------ ---------- ---------- ---------- ---------- ---------- ---------- ---------- ---------- ---------- ---------- [ 480]

#Thai-408 IAAEVAKAEK AKIE------ ---------- ---------- ---------- ---------- ---------- ---------- ---------- ---------- ---------- ---------- [ 480]

#India-IN1 IAAEVAKAEK AKIE------ ---------- ---------- ---------- ---------- ---------- ---------- ---------- ---------- ---------- ---------- [ 480]

#Thai-TB123 IAAEVAKAEK AKLE------ ---------- ---------- ---------- ---------- ---------- ---------- ---------- ---------- ---------- ---------- [ 480]

#Thai-T139 IAAEVAKAEK AKIE------ ---------- ---------- ---------- ---------- ---------- ---------- ---------- ---------- ---------- ---------- [ 480]

#Thai-TF127 IAAEVAKAEK AKLE------ ---------- ---------- ---------- ---------- ---------- ---------- ---------- ---------- ---------- ---------- [ 480]

#Thai-104 IAAEVAKAEK AKLE------ ---------- ---------- ---------- ---------- ---------- ---------- ---------- ---------- ---------- ---------- [ 480]

#Thai-413 IAAEVAKAEK AKLE------ ---------- ---------- ---------- ---------- ---------- ---------- ---------- ---------- ---------- ---------- [ 480]

#Thai-431 IAAEVAKAEK AKLE------ ---------- ---------- ---------- ---------- ---------- ---------- ---------- ---------- ---------- ---------- [ 480]

#Thai-447 IAAEVAKAEK AKLE------ ---------- ---------- ---------- ---------- ---------- ---------- ---------- ---------- ---------- ---------- [ 480]

#Thai-465 IAAEVAKAEK AKLE------ ---------- ---------- ---------- ---------- ---------- ---------- ---------- ---------- ---------- ---------- [ 480]

#Thai-425 IAAEVAKAEK AKLE------ ---------- ---------- ---------- ---------- ---------- ---------- ---------- ---------- ---------- ---------- [ 480]

#Thai-421 IAAEVAKAEK AKIE------ ---------- ---------- ---------- ---------- ---------- ---------- ---------- ---------- ---------- ---------- [ 480]

#Papau-PNN1 IAAEVAKAEK AKIE------ ---------- ---------- ---------- ---------- ---------- ---------- ---------- ---------- ---------- ---------- [ 480]

#China-CG15 IAAEVAKAEK AKIE------ ---------- ---------- ---------- ---------- ---------- ---------- ---------- ---------- ---------- ---------- [ 480]

#AY454087-Br67T IAAEVAKAEK AKIE------ ---------- ---------- ---------- ---------- ---------- ---------- ---------- ---------- ---------- ---------- [ 480]

#AY454088-Br781B IAAEVAKAEK AKLE------ ---------- ---------- ---------- ---------- ---------- ---------- ---------- ---------- ---------- ---------- [ 480]

#AY454090-EcuB IAAEVAKAEK AKIE------ ---------- ---------- ---------- ---------- ---------- ---------- ---------- ---------- ---------- ---------- [ 480]

#AY454094-Ong IAAEVAKAEK AKIE------ ---------- ---------- ---------- ---------- ---------- ---------- ---------- ---------- ---------- ---------- [ 480]

#AY454086-Br67B IAAEVAKAEK AKIE------ ---------- ---------- ---------- ---------- ---------- ---------- ---------- ---------- ---------- ---------- [ 480]

#Thai-105 IAVEVAKAEE AKKE------ ---------- ---------- ---------- ---------- ---------- ---------- ---------- ---------- ---------- ---------- [ 480]

#PVMG01384-Mauri IAAEVVKAEK ARIEAEEAEK EAGEAKTKTE ATEKEVLKIS DESKAAKVKK AVEKAKEAEK QAKSEAEKAK GMADDAGGKG TTNLEDVLTK LSEVLTSVKS LASNAEVASK NAKKEMTKAQ [ 480]

#PVIIG04181-Indi IAAEVAKAEK AKIE------ ---------- ---------- ---------- ---------- ---------- ---------- ---------- ---------- ---------- ---------- [ 480]

#PVBG05499-Brazi IAAEVAKAEK AKLE------ ---------- ---------- ---------- ---------- ---------- ---------- ---------- ---------- ---------- ---------- [ 480]

Insert A ><Conserved>< Insert B

#XM001613146-Sal IAAEVAKAEK AKIEAENAKL LADTASKAAE NIAKSSKAAK IANNVSTIAA EKSKVATEAA DEAAKALDET ENPESKIAEV TEKATKAVNA AEEAKKEKAK AEVAVEVAHA EVAKEKAQEA [ 600]

#AY454081-Br1T ---------- ----AENAKL IADTASKAAE DIAKSSKAAQ IAKNVSAKAE EKSKVATEAA DEAANALNEA ENPESKIDDV KKKATEAVNA AEEAKKEKSK AEIAVEVAKA EEAKKEAGKA [ 600]

#AY454082-Br69 ---------- ----AENAKL LADTASKAAE NIAKSSKAAK IANNVSTIAA EKSKVATEAA DEAAKALDET ENPESKIAEV TEKATKAVNA AEEAKKEKAK AEVAVEVAHA EVAKEKAQEA [ 600]

#AY454092-India ---------- ----AENAKL IADTASKAAE DIAKSSKAAQ IAKNVSAKAE EKSKVATEAA DEAANALNEA ENPESKIDDV RKKATEAVNA AEEAKKEKSK AEIAVEVAKA EEAKKEAGKA [ 600]

#AY454091-EcuT ---------- ----AENANL LADTANKEVE RIAKSSKAAQ IANKVSSKAA GKLEVATKAE DEAAKALDET ENSESKIAEV REKATTAFNA AEEAKKEK-- ---------- ---------- [ 600]

#Thai-414 ---------- ----AENANL LADTANKEVE RIAKSSKAAQ IANKVSSKAA GKLEVATKAE DEAAKALDEA ENSESKIAEV REKATTAFNA AEEAKKEK-- ---------- ---------- [ 600]

#Thai-129 ---------- ----AENANL LADAANKEVE RIAKSSNAAQ IANKVSSKAA GKLEVATKAE DEAAKALDET ENSESKIAEV REKATTAFNA AEEAKKEK-- ---------- ---------- [ 600]

#Thai-T120 ---------- ----AENANL LADTANKEVE RIAKSSKAAQ IANKVSSHAA GKLEVATKAE DEAAKALDET ENSESKIAEV REKATTAFNA AEEAKKEK-- ---------- ---------- [ 600]

#Thai-462 ---------- ----AENANL LADTANKEVE RIAKSSKAAQ IANKLSSKAA GKLEVATKAE DEAAKALDET ENSESKIAEV REKATTAFNA AEEAHKEK-- ---------- ---------- [ 600]

#China-CG12 ---------- ----AENANL LADTANKEVE RIAKSSKAAQ IANKVSSKAA GKLEVATKAE DEAAKALDET ENSESKIAEV REKATTAFNA AEEAKKEK-- ---------- ---------- [ 600]

#China-CG8 ---------- ----AENAKL LA-------- ---------- ---------- ---------- ---------- ---------- ---------- ---------- ---------- ---------- [ 600]

#AY454084-Bangl ---------- ----AENAKL LAETANKEVQ NIQKSSKAAQ IANNVSAKAS EKSKVATQAA DEAAKALEEV SKELEKAENP ESKIAEVKDK ATKAFDAAEV AK-------- ---------- [ 600]

#AY454097-SL57 ---------- ----AENAKL LADTASKAAE NIAKSSKAAK IANNVSTIAA EKSKVATEAA DEAAKALDET ENPESKIAEV TEKATKAVNA AEEAK----- ---------- ---------- [ 600]

#AY454096-SL ---------- ----AENAKL LADTASKAAE NIAKSSKAAK IANNVSTIAA EKSKVATEAA DEAAKALDET ENPESKIAEV TEKATKAVNA AEEAK----- ---------- ---------- [ 600]

#Thai-106 ---------- ----AENAKL LAETANKEVQ NIQKSSKAAQ IANNVSAKAS EKSKVATQAA DEAAKALEEV SKELEKAENP ESKIAEVKDK TTKAFDAAEV AK-------- ---------- [ 600]

#Thai-NR1 ---------- ----AENAKL LAETANKEVQ NIQKSSKAAQ IANNVSAKAS EKSKVATQAA DEAAKALEEV SKELEKAENP ESKIAEVKDK ATKAFDAAEV AK-------- ---------- [ 600]

#Thai-T107 ---------- ----AENAKL LAETANKEVQ NIQKSSKAAQ IANNVSAKAS EKSKVATQAA DEAAKALEEV SKELEKAENP ESKIAEVKDK ATKAFDAAEV AK-------- ---------- [ 600]

#Thai-113 ---------- ----AENAKL LAETANKEVQ NIQKSSKAAQ IANNVSAKAS EKSKVATQAA DEAAKALEEV SKELEKAENP ESKIAEVKDK ATKAFDAAEV AK-------- ---------- [ 600]

#Thai-430 ---------- ----AENAKL LAETANKEVQ NIQKSSKAAQ IAKNVSAKAE EKSKVATQAA DEAANALNEA ENPESKIDDV KKKATEAVNA AEEAK----- ---------- ---------- [ 600]

#China-CG11 ---------- ----AENAKL LAETANKEVQ NIQKSSKAAQ IANNVSAKAS EKSKVATQAA DEAAKALEEV SKELEKAENP ESKIAEVKDK ATKAFDAAEV AK-------- ---------- [ 600]

#AY454083-Chess IAAEVAKAGK AKIEAENANF LA-------- ---------- ---------- ---------- ---------- ---------- ---------- ---------- ---------- ---------- [ 600]

#Thai-TC103 IAAEVAKAEK AKIEAENAKL LA-------- ---------- ---------- ---------- ---------- ---------- ---------- ---------- ---------- ---------- [ 600]

#Thai-TV400 IAAEVAKAGK AKIEAENANF LA-------- ---------- ---------- ---------- ---------- ---------- ---------- ---------- ---------- ---------- [ 600]

#AY454089-Br781T IAAEVAKAEK AKIEAENAKL LA-------- ---------- ---------- ---------- ---------- ---------- ---------- ---------- ---------- ---------- [ 600]

#PVNG01493-NK IAAEVAKAEK AKIEAENAKL LA-------- ---------- ---------- ---------- ---------- ---------- ---------- ---------- ---------- ---------- [ 600]

#Thai-411 ---------- ----AENAKL LA-------- ---------- ---------- ---------- ---------- ---------- ---------- ---------- ---------- ---------- [ 600]

#Thai-TF83 IAAEVAKAEK AKIEAENAKL LA-------- ---------- ---------- ---------- ---------- ---------- ---------- ---------- ---------- ---------- [ 600]

#Thai-109 IAAEVAKAEK AKIEAENAKL LA-------- ---------- ---------- ---------- ---------- ---------- ---------- ---------- ---------- ---------- [ 600]

#Thai-410 IAAEVAKAEK AKIEAENAKL LA-------- ---------- ---------- ---------- ---------- ---------- ---------- ---------- ---------- ---------- [ 600]

#AY454098-Thai IAAEVAKAEK AKIEAENAKL LA-------- ---------- ---------- ---------- ---------- ---------- ---------- ---------- ---------- ---------- [ 600]

#AF099662-Belem ---------- ----AENANF LA-------- ---------- ---------- ---------- ---------- ---------- ---------- ---------- ---------- ---------- [ 600]

#AY454080-Br1B ---------- ----AENANF LA-------- ---------- ---------- ---------- ---------- ---------- ---------- ---------- ---------- ---------- [ 600]

#AY454085-Br56 ---------- ----AENANF LA-------- ---------- ---------- ---------- ---------- ---------- ---------- ---------- ---------- ---------- [ 600]

#Thai-115 ---------- ----AENANL LA-------- ---------- ---------- ---------- ---------- ---------- ---------- ---------- ---------- ---------- [ 600]

#Thai-TF91 ---------- ----AENANF LA-------- ---------- ---------- ---------- ---------- ---------- ---------- ---------- ---------- ---------- [ 600]

#Thai-126 ---------- ----AENANF LA-------- ---------- ---------- ---------- ---------- ---------- ---------- ---------- ---------- ---------- [ 600]

#Thai-442 ---------- ----AENANL LA-------- ---------- ---------- ---------- ---------- ---------- ---------- ---------- ---------- ---------- [ 600]

#Thai-408 ---------- ----AENANF LA-------- ---------- ---------- ---------- ---------- ---------- ---------- ---------- ---------- ---------- [ 600]

#India-IN1 ---------- ----AENANF LA-------- ---------- ---------- ---------- ---------- ---------- ---------- ---------- ---------- ---------- [ 600]

#Thai-TB123 ---------- ----AENANL LA-------- ---------- ---------- ---------- ---------- ---------- ---------- ---------- ---------- ---------- [ 600]

#Thai-T139 ---------- ----AENAKL LA-------- ---------- ---------- ---------- ---------- ---------- ---------- ---------- ---------- ---------- [ 600]

#Thai-TF127 ---------- ----AENANF LA-------- ---------- ---------- ---------- ---------- ---------- ---------- ---------- ---------- ---------- [ 600]

#Thai-104 ---------- ----AENANF LA-------- ---------- ---------- ---------- ---------- ---------- ---------- ---------- ---------- ---------- [ 600]

#Thai-413 ---------- ----AENANL LA-------- ---------- ---------- ---------- ---------- ---------- ---------- ---------- ---------- ---------- [ 600]

#Thai-431 ---------- ----AENANL LA-------- ---------- ---------- ---------- ---------- ---------- ---------- ---------- ---------- ---------- [ 600]

#Thai-447 ---------- ----AENANL LA-------- ---------- ---------- ---------- ---------- ---------- ---------- ---------- ---------- ---------- [ 600]

#Thai-465 ---------- ----AENANL LA-------- ---------- ---------- ---------- ---------- ---------- ---------- ---------- ---------- ---------- [ 600]

#Thai-425 ---------- ----AENANF LA-------- ---------- ---------- ---------- ---------- ---------- ---------- ---------- ---------- ---------- [ 600]

#Thai-421 ---------- ----AENAKL LA-------- ---------- ---------- ---------- ---------- ---------- ---------- ---------- ---------- ---------- [ 600]

#Papau-PNN1 ---------- ----AENANF LA-------- ---------- ---------- ---------- ---------- ---------- ---------- ---------- ---------- ---------- [ 600]

#China-CG15 ---------- ----AENANF LA-------- ---------- ---------- ---------- ---------- ---------- ---------- ---------- ---------- ---------- [ 600]

#AY454087-Br67T ---------- ----AENANF LA-------- ---------- ---------- ---------- ---------- ---------- ---------- ---------- ---------- ---------- [ 600]

#AY454088-Br781B ---------- ----AENANL LA-------- ---------- ---------- ---------- ---------- ---------- ---------- ---------- ---------- ---------- [ 600]

#AY454090-EcuB ---------- ----AENANF LA-------- ---------- ---------- ---------- ---------- ---------- ---------- ---------- ---------- ---------- [ 600]

#AY454094-Ong ---------- ----AENANF LA-------- ---------- ---------- ---------- ---------- ---------- ---------- ---------- ---------- ---------- [ 600]

#AY454086-Br67B ---------- ----AENANF LA-------- ---------- ---------- ---------- ---------- ---------- ---------- ---------- ---------- ---------- [ 600]

#Thai-105 ---------- ----AGKAKV AAKQVADKSK LEKAIQAADK ASKKTDEASK LAEEALSDLE SLEKETGEIK TKVNEIKEKV QNAINAALEA HKEKTIAEIT VEVAMAEEAK KEADNAKVAA [ 600]

#PVMG01384-Mauri IAAEVAKAEK AKIEAENAKL LA-------- ---------- ---------- ---------- ---------- ---------- ---------- ---------- ---------- ---------- [ 600]

#PVIIG04181-Indi ---------- ----AENANF LA-------- ---------- ---------- ---------- ---------- ---------- ---------- ---------- ---------- ---------- [ 600]

#PVBG05499-Brazi ---------- ----AENANL LA-------- ---------- ---------- ---------- ---------- ---------- ---------- ---------- ---------- ---------- [ 600]

Insert B >< Dimorphic

#XM001613146-Sal KEAAKQVADK SKLEKAIQAA DKASEKANEA SKLAEEALSN LESLEKETGE IVEKVNAIEQ KVQTAKNAAI EAHKEKTKAE IAVEVAKAEE AKKEADNAKV AAEKAKETAE KIAKTSKSTE [ 720]

#AY454081-Br1T KVAAKQVADK SKLEKAIQAA DKASKKTDEA SKLAEEALSD LESLEKETGE IKTKVNEIKE KVQNAINAAL EAHKEKTIAE ITVEVAKAEE AKKEADNAKV AAEKAKETAE KIAKTSKSTE [ 720]

#AY454082-Br69 KEAAKQVADK SKLEKAIQAA DKASEKANEA SKLAEEALSN LESLEKETGE IVEKVNAIEQ KVQTAKNAAI EAHKEKTKAE IAVEVAKAEE AKKEADNAKV AAEKAKETAE KIAKTSKSTE [ 720]

#AY454092-India KVAAKQVADK SKLEKAIQAA DKASKKTDEA SKLAEEALSD LESLEKETGE IKTKVNEIKE KVQNAINAAL EAHKEKTIAE ITVEVAKAEE AKKEADNAKV AAEKAKETAE KIAKTSKSTE [ 720]

#AY454091-EcuT ---------- ---------- ---------- ---------- ---------- ---------- ---------- ------TKAK IAVEVAKAEE AKKEADNAKI AAEKAKQTAE KIAKTSKSTE [ 720]

#Thai-414 ---------- ---------- ---------- ---------- ---------- ---------- ---------- ------TKAK IAVEVAKAEE AKKEADNAKI AAEKAKQTAE KIAKTSKSTE [ 720]

#Thai-129 ---------- ---------- ---------- ---------- ---------- ---------- ---------- ------TKAK IAVEVAKAEE AKKEADNAKI AAEKAKQTAE KIAKTSKSTE [ 720]

#Thai-T120 ---------- ---------- ---------- ---------- ---------- ---------- ---------- ------TKAK IAVEVAKAEE AKKEADNAKI AAEKAKQTAE KIAKTSKSTE [ 720]

#Thai-462 ---------- ---------- ---------- ---------- ---------- ---------- ---------- ------TKAE IAVEVAKAKE AKKEADNAKV AAEKAKQTAE KIAKTSKSTE [ 720]

#China-CG12 ---------- ---------- ---------- ---------- ---------- ---------- ---------- ------TKAK IAVEVAKAEE AKKEADNAKI AAEKAKQTAE KIAKTSKSTE [ 720]

#China-CG8 ---------- ---------- ---------- ---------- ---------- ---------- ---------- ---------- ---------- ---------- --EKAKQTAE KIAKTSKSTE [ 720]

#AY454084-Bangl ---------- ---------- ---------- ---------- ---------- ---------- ---------- ---------- -------KEN AKAGVAVEVA HAEVAKGKAQ EAKEATKQVA [ 720]

#AY454097-SL57 ---------- ---------- ---------- ---------- ---------- ---------- ---------- ---------- -------KEK AKAEVAVEVA HAEVAKEKAQ EAKEAAKQVA [ 720]

#AY454096-SL ---------- ---------- ---------- ---------- ---------- ---------- ---------- ---------- -------KEK AKAEVAVEVA HAEVAKEKAQ EAKEAAKQVA [ 720]

#Thai-106 ---------- ---------- ---------- ---------- ---------- ---------- ---------- ---------- -------KEN AKAGVAVEVA HAEVAKGKAQ EAKEATKQVA [ 720]

#Thai-NR1 ---------- ---------- ---------- ---------- ---------- ---------- ---------- ---------- -------KEN AKAGVAVEVA HAEVAKGKAQ EAKEATKQVA [ 720]

#Thai-T107 ---------- ---------- ---------- ---------- ---------- ---------- ---------- ---------- -------KEN AKAGVAVEVA HAEVAKGKAQ EAKEATKQVA [ 720]

#Thai-113 ---------- ---------- ---------- ---------- ---------- ---------- ---------- ---------- -------KEN AKAGVAVEVA HAEVAKGKAQ EAKEATKQVA [ 720]

#Thai-430 ---------- ---------- ---------- ---------- ---------- ---------- ---------- ---------- -------KEK SKAEIAVEVA KAEEAKKEAG KAKVAAKQVA [ 720]

#China-CG11 ---------- ---------- ---------- ---------- ---------- ---------- ---------- ---------- -------KEN AKAGVAVEVA HAEVAKGKAQ EAKEATKQVA [ 720]

#AY454083-Chess ---------- ---------- ---------- ---------- ---------- ---------- ---------- ---------- ---------- ---------- --EKAKQTAE KIAKTSKSTE [ 720]

#Thai-TC103 ---------- ---------- ---------- ---------- ---------- ---------- ---------- ---------- ---------- ---------- --EKAKETAE KIAKTSKSTE [ 720]

#Thai-TV400 ---------- ---------- ---------- ---------- ---------- ---------- ---------- ---------- ---------- ---------- --EKAKQTAE KIAKTSKSTE [ 720]

#AY454089-Br781T ---------- ---------- ---------- ---------- ---------- ---------- ---------- ---------- ---------- ---------- --EKAKETAE KIAKASKSTE [ 720]

#PVNG01493-NK ---------- ---------- ---------- ---------- ---------- ---------- ---------- ---------- ---------- ---------- --EKAKETAE KIAKASKSTE [ 720]

#Thai-411 ---------- ---------- ---------- ---------- ---------- ---------- ---------- ---------- ---------- ---------- --EKAKETAE KIAKASKSTE [ 720]

#Thai-TF83 ---------- ---------- ---------- ---------- ---------- ---------- ---------- ---------- ---------- ---------- --EKAKETAE KIAKASKSTE [ 720]

#Thai-109 ---------- ---------- ---------- ---------- ---------- ---------- ---------- ---------- ---------- ---------- --EKAKETAE KIAKSSKSTE [ 720]

#Thai-410 ---------- ---------- ---------- ---------- ---------- ---------- ---------- ---------- ---------- ---------- --EKAKETAE KIAKASKSTE [ 720]

#AY454098-Thai ---------- ---------- ---------- ---------- ---------- ---------- ---------- ---------- ---------- ---------- --EKAKETAE KIAKASKSTE [ 720]

#AF099662-Belem ---------- ---------- ---------- ---------- ---------- ---------- ---------- ---------- ---------- ---------- --EKAKQTAE KIAKTSKSTE [ 720]

#AY454080-Br1B ---------- ---------- ---------- ---------- ---------- ---------- ---------- ---------- ---------- ---------- --EKAKQTAE KIAKTSKSTE [ 720]

#AY454085-Br56 ---------- ---------- ---------- ---------- ---------- ---------- ---------- ---------- ---------- ---------- --EKAKQTAE KIAKTSKSTE [ 720]

#Thai-115 ---------- ---------- ---------- ---------- ---------- ---------- ---------- ---------- ---------- ---------- --EKAKQTAE KIAKTSKSTE [ 720]

#Thai-TF91 ---------- ---------- ---------- ---------- ---------- ---------- ---------- ---------- ---------- ---------- --EKAKQTAE KIAKTSKSTE [ 720]

#Thai-126 ---------- ---------- ---------- ---------- ---------- ---------- ---------- ---------- ---------- ---------- --EKAKQTAE KIAKTSKSTE [ 720]

#Thai-442 ---------- ---------- ---------- ---------- ---------- ---------- ---------- ---------- ---------- ---------- --EKAKQTAE KIAKTSKSTE [ 720]

#Thai-408 ---------- ---------- ---------- ---------- ---------- ---------- ---------- ---------- ---------- ---------- --EK--QTAE KIAKTSKSTE [ 720]

#India-IN1 ---------- ---------- ---------- ---------- ---------- ---------- ---------- ---------- ---------- ---------- --EKAKQTAE KIAKTSKSTE [ 720]

#Thai-TB123 ---------- ---------- ---------- ---------- ---------- ---------- ---------- ---------- ---------- ---------- --EKAKQTAE KIAKTSKSTE [ 720]

#Thai-T139 ---------- ---------- ---------- ---------- ---------- ---------- ---------- ---------- ---------- ---------- --EKAKQTAE KIAKTSKSTE [ 720]

#Thai-TF127 ---------- ---------- ---------- ---------- ---------- ---------- ---------- ---------- ---------- ---------- --EKAKQTAE KIAKTSKSTE [ 720]

#Thai-104 ---------- ---------- ---------- ---------- ---------- ---------- ---------- ---------- ---------- ---------- --EKAKQPAE KIAKTSKSPE [ 720]

#Thai-413 ---------- ---------- ---------- ---------- ---------- ---------- ---------- ---------- ---------- ---------- --EKAKQTAE KIAKTSKSTE [ 720]

#Thai-431 ---------- ---------- ---------- ---------- ---------- ---------- ---------- ---------- ---------- ---------- --EKAKQTAE KIAKTSKSTE [ 720]

#Thai-447 ---------- ---------- ---------- ---------- ---------- ---------- ---------- ---------- ---------- ---------- --EKAKQTAE KIAKTSKSTE [ 720]

#Thai-465 ---------- ---------- ---------- ---------- ---------- ---------- ---------- ---------- ---------- ---------- --EKAKQTAE KIAKTSKSTE [ 720]

#Thai-425 ---------- ---------- ---------- ---------- ---------- ---------- ---------- ---------- ---------- ---------- --EKAKQTAE KIAKTSKSTE [ 720]

#Thai-421 ---------- ---------- ---------- ---------- ---------- ---------- ---------- ---------- ---------- ---------- --EKAKQTAE KIAKTSKSTE [ 720]

#Papau-PNN1 ---------- ---------- ---------- ---------- ---------- ---------- ---------- ---------- ---------- ---------- --EKAKQTAE KIAKTSKSTE [ 720]

#China-CG15 ---------- ---------- ---------- ---------- ---------- ---------- ---------- ---------- ---------- ---------- --EKAKQTAE KIAKTSKSTE [ 720]

#AY454087-Br67T ---------- ---------- ---------- ---------- ---------- ---------- ---------- ---------- ---------- ---------- --EKAKQTAE KIAKTSKSTE [ 720]

#AY454088-Br781B ---------- ---------- ---------- ---------- ---------- ---------- ---------- ---------- ---------- ---------- --EKAKQTAE KIAKTSKSTE [ 720]

#AY454090-EcuB ---------- ---------- ---------- ---------- ---------- ---------- ---------- ---------- ---------- ---------- --EKAKQTAE KIAKTSKSTE [ 720]

#AY454094-Ong ---------- ---------- ---------- ---------- ---------- ---------- ---------- ---------- ---------- ---------- --EKAKQTAE KIAKTSKSTE [ 720]

#AY454086-Br67B ---------- ---------- ---------- ---------- ---------- ---------- ---------- ---------- ---------- ---------- --EKAKQTAE KIAKTSKSTE [ 720]

#Thai-105 ---------- ---------- ---------- ---------- ---------- ---------- ---------- ---------- ---------- ---------- --ERAKEAAE KIAKTSKSTE [ 720]

#PVMG01384-Mauri ---------- ---------- ---------- ---------- ---------- ---------- ---------- ---------- ---------- ---------- ---KAKETAE KIAKASKSTE [ 720]

#PVIIG04181-Indi ---------- ---------- ---------- ---------- ---------- ---------- ---------- ---------- ---------- ---------- ---KAKQTAE KIAKTSKSTE [ 720]

#PVBG05499-Brazi ---------- ---------- ---------- ---------- ---------- ---------- ---------- ---------- ---------- ---------- ---KAKQTAE KIAKTSKSTE [ 720]

Dimorphic >< Conserved

#XM001613146-Sal --KITEEVRK ATEFAKTAGD ETTLAATKAE SEIPSEEKNQ KELLDSIKQK AESAFQASQE AIKAKTEAEN FLEIAKEVPK AEAAKEEAQK AATAAEEAKT EVLKIAEEVN KSDASESEKK [ 840]

#AY454081-Br1T --KITEEVRK ATEFAKTAGD ATTQAATEAA GDVSSEEQNQ KKMLQSIKQK AESALEASQE AIKAKTEAEN FLEIAKEVPK AEAAKEEAQK AATAAEEAKT EALKIAEEVN KSDASESEKK [ 840]

#AY454082-Br69 --KITEEVRK ATEFAKTAGD ETTLAATKAE SEIPSEEKNQ KELLDSIKQK AESAFQASQE AIKAKTEAEN FLEIAKEVPK AEAAKEEAQK AATAAEEAKT EALKIAEEVN KSDASESEKK [ 840]

#AY454092-India --KITEEVRK ATEFAKTAGD ATTQAATEAA GDVSSEEQKQ KVLLESIKQK AESAFQASQE AIKAKTEAEN FLEIAKEVPK AEAAKEEAQK AATAAEEAKT EALKIAEEVN KSDASESEKK [ 840]

#AY454091-EcuT --KIIEAVTK ATKFAKTAGD ETTLAATKAA SEVPSEEEKQ KELLESIKEK AESALQASQE AIKAKTEAEN FLEIAKEVPK AEAAKEEAEK AATAAEEAKT EALKIAEEVN KSDASESEKK [ 840]

#Thai-414 --KIIEAVTK ATKFAKTAGD ETTLAATKAA SEVPSEEEKQ KELLESIKEK AESALQASQE AIKAKTEAEN FLEIAKEVPK AEAAKEEAEK AATAAEEAKT EALKIAEEVN KSDASESEKK [ 840]

#Thai-129 --KIIEAVTK ATKFAETAGD ETTLAATKAA SEVPSEEEKQ KELLESIKEK AESALQASQE AIKAKTEAEN FLEIAKEVPK AEAAKEEAEK AATAAEEAKT EALKIAEEVN KSDASESEKK [ 840]

#Thai-T120 --KIIEAVTK ATKFAKTAGD ETTLAATKAA SEVPSEEEKQ KELLESIKEK AESALQASQE AIKAKTEAEN FLEIAKEVPK AEAAKEEAEK AATAAEEAKT EALKIAEEVN KSDASESEKK [ 840]

#Thai-462 --KIIEAVTK ATKFAKTAGD ETIETEKKAA GEVPSEEKNQ KELLDSIKQK AESALQASQE AIKAKTEAEK FLEIAKEVPK AEAAKEEAQK AATSADEAKT EALKIAEEVN KSDASESEKK [ 840]

#China-CG12 --KIIEAVTK ATKFAKTAGD ETTLAATKAA SEVPSEEEKQ KELLESIKEK AESALQASQE AIKAKTEAEN FLEIAKEVPK AEAAKEEAEK AATAAEEAKT EALKIAEEVN KSDASESEKK [ 840]

#China-CG8 --KIIEAVTK ATKFAKTAGD ETTLAATKAA SEVPSEEEKQ KELLESIKEK AESALQASQE AIKAKTEAEN FLEIAKEVPK AEAAKEEAEK AATAAEEAKT EALKIAEEVN KSDASESEKK [ 840]

#AY454084-Bangl DKSKLEKAIQ AADKASENAD EASKLANEAL SDLESLENET GEIVAKVNEI KQKVQKAKNA AIEAHKEKT- KAEIAVEVAK AEEAKKEADK AKVAAEKAKT EALKIAEEVN KSDASESEKK [ 840]

#AY454097-SL57 DKSKLEKAIQ AADKASEKAN EASKLAEEAL SNLESLEKET GEIVEKVNAI KQKVQKAKNA AIEAHKEKT- KAEIAVEVAK AEEAKKEADK AKVAAEKAKT EALKIAEEVN KSDASESEKK [ 840]

#AY454096-SL DKSKLEKAIQ AADKASEKAN EASKLAEEAL SNLESLEKET GEIVEKVNAI KQKVQKAKNA AIEAHKEKT- KAEIAVEVAK AEEAKKEADK AKVAAEKAKT EALKIAEEVN KSDASESEKK [ 840]

#Thai-106 DKSKLEKAIQ AADKASENAD EASKLANEAL SDLESLENET GEIVAKVNEI KQKVQKAKNA AIEAHKEKT- KAEIAVEVAK AEEAKKEADK AKVAAEKAKT EALKIAEEVN KSDASESEKK [ 840]

#Thai-NR1 DKSKLEKAIQ AADKASENAD EASKLANEAL SDLESLENET GEIVAKVNEI KQKVQKAKNA AIEAHKEKT- KAEIAVEVAK AEEAKKEADK AKVAAEKAKT EALKIAEEVN KSDASESEKK [ 840]

#Thai-T107 DKSKLEKAIQ AADKASENAD EASKLANEAL SDLESLENET GEIVAKVNEI KQKVQKAKNA AIEAHKEKT- KAEIAVEVAK AEEAKKEADK AKVAAEKAKT EALKIAEEVN KSDASESEKK [ 840]

#Thai-113 DKSKLEKAIQ AADKASENAD EASKLANGAL SDLESLENET GEIVAKVNEI KQKVQKAKNA AIEAHKEKT- KAEIAVEVAK AEEAKKEADK AKVAAEKAKT EALKIAEEVN KSDASESEKK [ 840]

#Thai-430 DKSKLEKAIQ AADKASENAD EASKLANEAL SDLESLENET GEIVAKVNEI KQKVQKANNA AIEAHKEKT- KAEIAVEVAK AEEAKKEADK VKVAAEKAKT EALKIAEEVN KSDASESEKK [ 840]

#China-CG11 DKSKLEKAIQ AADKASENAD EASKLANEAL SDLESLENET GEIVAKVNEI KQKVQKAKNA AIEAHKEKT- KAEIAVEVAK AEEAKKEADK AKVAAEKAKT EALKIAEEVN KSDASESEKK [ 840]

#AY454083-Chess --KIIEAVRK VTEFANKAGD ETTQATKEAE GEISSVEQNQ KKMLQSIKQK AESALEASQE AIKAKTEAEN FLEIAKEVPK AEAAKEEAQK AATAAEEAKT EALKIAEEVN KSDASESEKK [ 840]

#Thai-TC103 --KITEEVRK ATEFAKTARD ATTQAAKEAE GEVSLEEQKQ KDLLQSIKEK AESALQASKD AIKAKTEAEN FLEIAKEVPK AEAAKEEAEK AATEADEAKT EALKIAEEVK KSETSESEKK [ 840]

#Thai-TV400 --KIIEAVTK ATKFAKTAGD ETTLAATKAA SEVPSEEEKQ KELLESIKEK AESALQASQE AIKAKTEAEN FLEIAKEVPK AEAAKEEAQK AATAAEEAKT EALKIAEEVK KSEASESEKK [ 840]

#AY454089-Br781T --KITEEVIK ATKFAKTAGD ETTETETKAE GDVSLEEQNQ KDLLQSIKQK AESAFQASQE AIKAKTEAEN FLEIAKEVPK AEAAKEEAQK AATAAEEAKT EALKIAEEVN KSDASESEKK [ 840]

#PVNG01493-NK --KITEEVIK ATKFAKTAGD ETTETETKAE GDVSLEEQSQ KDLLQSIKQK AESALQASQE AIKAKTEAEN FLEIAKEVPK AEAAKEEAQK AATAAEEAKT EALKIAEEVN KSDASESEKK [ 840]

#Thai-411 --KITEEVIK APEFAKTAGD ETTLAATKAE SEIPSEEKNQ KELLDSIKQK AESAFQASQE AIKAKTEAEN FLEIAKEVPK AEAAKEEAQK AATAAEEAKT EALKIAEEVN KSDASESEKK [ 840]

#Thai-TF83 --KITEEVIK ATKFAKTAGD ETTETETKAE GDVSLEEQNQ KDLLQSIKQK AESAFQASQE AIKAKTEAEN FLEIAKEVPK AEAAKEEAQK AATAAEEAKT EALKIAEEVN KSDASESEKK [ 840]

#Thai-109 --KITEEVIK ATKFAKTAGD ETTETETKAE GDVSLEEQNQ KDLLQSIKQK AESALQASQE AIKAKTEAEN FPEIAKEVPK AGAAREEAQK AATAAEEAKT EALKIAEEVN KSDASESEKK [ 840]

#Thai-410 --KITEEVIK ATKFAKTAGD ETTETETKAE GDVSLEEQNQ KDLLQSIKQK AESAFQASQE AIKAKTEAEN FLEIAKGVPK AEAAKEEAQK AATAAEEAKT EALKIAEEVN KSDASESEKK [ 840]

#AY454098-Thai --KITEEVIK ATKFAKTAGD ETTETETKAE GDVSLEEQNQ KDLLQSIKQK AESALQASQE AIKAKTEAEN FLEIAKEVPK AEAAKEEAQK AATAAEEAKT EALKIAEEVN KSDASESEKK [ 840]

#AF099662-Belem --KITEEVRK ATEFAKTAGD ATTQAATEAA GDVSSEEQKQ KVLLESIKQK AESALQASKD AIKAKTEAEN FLEIAKEVPK AEAAKEEAQK AATSADEAKT EALKIAEEVN KSDASENEKK [ 840]

#AY454080-Br1B --KITEEVRK ATEFAKTAGD ATTQAATEAA GDVSSEEQKQ KVLLESIKQK AESALQASKD AIKAKTEAEN FLEIAKEVPK AEAAKEEAQK AATSADEAKT EALKIAEEVN KSDASENEKK [ 840]

#AY454085-Br56 --KITEEVRK ATEFAKTAGD ATTQAAKEAE GDVSLEEQNQ KDLLQSIKQK AESALQASQE AIKAKTEAEN FLEIAKEVPK AEAAKEEAQK AATAAEEAKT EALKIAEEVN KSDASESEKK [ 840]

#Thai-115 --KITEEVRK ATEFAKTAGD ATTQAATEAA GDVSSEEQKQ KVLLESIKQK AESALQASKD AIKAKTEAEN FLEIAKEVPK AEAAKEEAQK AATSADEAKT EALKIAEEVN KSDASENEKK [ 840]

#Thai-TF91 --KITEEVRK ATEFAKTAGD ATTQAATEAA GDVSSEEQKQ KVLLESIKQK AESALQASKD AIKAKTEAEN FLEIAKEVPK AEAAKEEAQK AATSADEAKT EALKIAEEVN KSDASENEKK [ 840]

#Thai-126 --KIIEAVTK ATKFAKTAGD ETTQAATKAA SEVPSEEEKQ KELLESVKEK AESALQASQE AIKAKTEAEN FLEIAKEVPK AEAAKEEAEK AATAAEEAKT EALKIAEEVN KSDASESEKK [ 840]

#Thai-442 --KITEEVRK ATEFAKTAGD ATTQAATEAA GDVSSEEQKQ KVLPESIKQK AESALQASKD AIKAKTEAEN FLEIAKEVPK AEAAKEEAQK AATSADEAKT EALKIAEEVN KSDASENEKK [ 840]

#Thai-408 --KITEEVRR ATEFAKTAGD ATTQAATEAA GDVSSEEQKQ KVLLESIQQK AESALQASKD AIKAKTEAEN FLEIAKEVPK AEAAKEEAQK AATSADEAKT EALKIAEEVN KSDASENEKK [ 840]

#India-IN1 --KITEEVRK ATEFAKTAGD ATTQAATEAA GDVSSEEQKQ KVLLESIKQK AESALQASKD AIKAKTEAEN FLEIAKEVPK AEAAKEEAQK AATSADEAKT EALKIAEEVN KSDASENEKK [ 840]

#Thai-TB123 --KITEEVRK ATEFAKTAGD ATTQAAKEAE GDVSLEEQNQ KDLLQSIKQK AESALQASQE AIKAKTEAEN FLEIAKEVPK AEAAKEEAQK AATAAEEAKT EALKIAEEVN KSDASESEKK [ 840]

#Thai-T139 --KITEEVRK ATEFAKTAGD ATTQAAKEAE GDVSLEEQNQ KDLLQSIKQK AESALQASQE AIKAKTEAEN FLEIAKEVPK AEAAKEEAQK AATAAEEAKT EALKIAEEVN KSDASESEKK [ 840]

#Thai-TF127 --KITEEVRK ATEFAKTAGD ATTQAAKEAE GDVSLEEQNQ KDLLQSIKQK AESALQASQE AIKAKTEAEN FLEIAKEVPK AEAAKKEAQK AATAAEEAKT EALKIAEEVN KSDASESEKK [ 840]

#Thai-104 --KITVEVRK ATEFAKTAGD ATTQAAKEAE GDVSLEEQNQ KDLLQSIKQK AESALQASQE AIKAKTEAEN FLEIAKEVPK AEAAKEEAQK AATAAEEAKT EALKIAEEVN KSDASESEKK [ 840]

#Thai-413 --KITEEVRK ATEFARTAGD ATTQAAKEAE GDVSLEEQNQ KDLLQSIKQK AESALQASQE AIKAKTEAEN FLEIAKEVPK AEAAKEEAQK AATAAEEAKT EALKIAEEVN KSDASESEKK [ 840]

#Thai-431 --KITEEVRK ATEFAKTAGD ATTQAAKEAE GDVSLEEQNQ KDLLQSIKQK AESALQASQE AIKAKTEAEN FLEIAKEVPK AEAAKEEAQE AATAAEEAKT EALKIAEEVN KSDASESEKK [ 840]

#Thai-447 --KITEEVRK ATEFAKTAGD ATTQAAKEAE GDVSLEEQNQ KDLLQSIKQK AESALQASQE VIKAKTEAEN FLEIAKEVPK AEAAKEEAQK AATAAEEAKT EALKIAEEVN KSDASESEKK [ 840]

#Thai-465 --KITEEVRK ATEFAKTAGD ATTQAAKEAE GDVSLEEQNQ KDLLQSIKQK AESALQASQE VIKAKTEAEN FLEIAKEVPK AEAAKEEAQK AATAAEEAKT EALKIAEEVN KSDAPESEKK [ 840]

#Thai-425 --KITEEVRK ATEFAKTAGD ATTQAAKEAE GDVSLEEQNQ KDLLQSIKQK AESASQASQE AIKAKTEAEN FLEIAKEVPK AEAAKEEAQK AATAAEEAKT EALKIAEEVN KSDASESEKK [ 840]

#Thai-421 --KITEEVRK ATEFAKTAGD ATTQAAKEAE GDVSLEEQNQ KDLLQSIKQK GESALQASQE AIKAKTEAEN FLEIAKEVPK AEAAKEEAQK AATAAEEAKT EALKIAEEVN KSDASESEKK [ 840]

#Papau-PNN1 --KITEEVRK ATEFAKTAGD ATTQAAKEAE GDVSLEEQNQ KDLLQSIKQK AESALQASQE AIKAKTEAEN FLEIAKEVPK AEAAKEEAQK AATAAEEAKT EALKIAEEVN KSDASESEKK [ 840]

#China-CG15 --KITEEVRK ATEFAKTAGD ATTQAATEAA GDVSSEEQKQ KVLLESIKQK AESALQASKD AIKAKTEAEN FLEIAKEVPK AEAAKEEAQK AATSADEAKT EALKIAEEVN KSDASENEKK [ 840]

#AY454087-Br67T --KITEEVRK ATEFAKTAGD ATTQAAKEAE GDVSLEEQNQ KDLLQSIKQK AESALQASQE AIKAKTEAEN FLEIAKEVPK AEAAKEEAQK AATAAEEAKT EALKIAEEVN KSDASESEKK [ 840]

#AY454088-Br781B --KITEEVRK ATEFAKTAGD ATTQAAKEAE GDVSLEEQNQ KDLLQSIKQK AESALQASQE AIKAKTEAEN FLEIAKEVPK AEAAKEEAQK AATAAEEAKT EALKIAEEVN KSDASESEKK [ 840]

#AY454090-EcuB --KITEEVRK ATEFAKTAGD ATTQAATEAA GDVSSEEQKQ KVLLESIKQK AESALQASKD AIKAKTEAEN FLEIAKEVPK AEAAKEEAQK AATSADEAKT EALKIAEEVN KSDASENEKK [ 840]

#AY454094-Ong --KITEEVRK ATEFAKTAGD ATTQAAKEAE GDVSLEEQNQ KDLLQSIKQK AESALQASQE AIKAKTEAEN FLEIAKEVPK AEAAKEEAQK AATAAEEAKT EALKIAEEVN KSDASESEKK [ 840]

#AY454086-Br67B --KITEEVRK ATEFAKTAGD ATTQAAKEAE GDVSLEEQNQ KDLLQSIKQK AESALQASQE AIKAKTEAEN FLEIA----- ---------- ---------- ---------- ---------- [ 840]

#Thai-105 --KITEEVRK ATEFAKTAGD ATTQAATEAA GDVSSEEQKQ KVLLESIKQK AESAFQASQE AVKAKTEAEN FLEIAKEVPK AEAAKEEAQK AATAAEEAKT EALKIAEEVN KSDASESEKK [ 840]

#PVMG01384-Mauri --KITEEVIK ATKFAKTAGD ETTETETKAE GDVSLEEQNQ KDLLQSIKQK AESAFQASQE AIKAKTEAEN FLEIAKEVPK AEAAKEEAQK AATAAEEAKT EALKIAEEVN KSDASESEKK [ 840]

#PVIIG04181-Indi --KITEEVRK ATEFAKTAGD ATTQAATEAA GDVSSEEQKQ KVLLESIKQK AESALQASKD AIKAKTEAEN FLEIAKEVPK AEAAKEEAQK AATSADEAKT EALKIAEEVN KSDASENEKK [ 840]

#PVBG05499-Brazi --KITEEVRK ATEFAKTAGD ATTQAAKEAE GDVSLEEQNQ KDLLQSIKQK AESALQASQE AIKAKTEAEN FLEIAKEVPK AEAAKEEAQK AATAAEEAKT EALKIAEEVN KSDASESEKK [ 840]

#XM001613146-Sal KIETAANETA GEAEKAATFA KEAADAAKDT NKAVTLAVAK EKVEKALKAA KEAKKANEKA SYALIRTKKQ YALEPLEITS EAGYNITEKE EQVKEEIEEQ DDKASEEEEE DTQQIDQTQI [ 960]

#AY454081-Br1T KIETAANETA GEAEKAATFA KEAADAAKDT NEAVTLAVAK EKVEKALKAA KEAKKANEKA SYALIRTKKQ YALEPLEITS EAGYNITEKE EQVKEEIEEQ EDKASEEEEE DTLQTDQTEI [ 960]

#AY454082-Br69 KIETAANETA GEAEKAATFA KEAADAAKDT NEAVTLAVAK EKVEKALKAA KEAKKANEKA SYALIRTKKQ YALEPLEITS EAGYNITEKE EQVKEEIEEQ KDKASEEEEE DTLQTDQTEI [ 960]

#AY454092-India KIETAANETV GEAEKAATFA KEAADAAKDT NEAVTLAVAK EKVEKALKAA KEAKKANEKA SYALIRTKKQ YALEPLEITS EAGYNITEKE EQVKEEIEEQ KDKASEEEEE DTLQTDQTEI [ 960]

#AY454091-EcuT KIETAANETA GEAEKAATFA KEAADAAKDT NEAVTLAVAK EKVEKALKAA KEAKKANEKA SYALIRTKKQ YALEPLEITS EAGYNITEKE EQVKEEIEEQ DDKASEEEEE DTLQIDNTEV [ 960]

#Thai-414 KIETAANETA GVAEKAATFA KEAADAAKDT NEAVTLAVAK EKVEKALKAA KEAKKANEKA SYALIRTKKQ YALEPLEITS EAGYNITEKE EQVKEEIEEQ DDKASEEEEE DTLQIDNTEV [ 960]

#Thai-129 KIETAANETA GEAQKAATFA KEAADAAKDT NEAVTLAVAK EKVEKALKAA KEAKKANEKA SYALIRTKKQ HALEPLEITS EAGYNITEKE EQVKEEIEEQ EDKASEEEEE DTLQTDQTEI [ 960]

#Thai-T120 KIETAANETA GEAEKAATFA KEAADAAKDT NEAVTLAVAK EKVEKALKAA KEAKKANEKA SYALIRTKKQ YALEPLEITS EAGYNITEKE EQVKEEIEEQ DDKASEEEEE DTLQIDNTEV [ 960]

#Thai-462 KIETEANATA GEAQKAATFA KEAADAAKDT NEAVTLVVAK EKVEKALKAA KEAKKASEKA SYALIRTKKQ YALEPLEITS EAGYNITEKE EQVKEEIEEQ DDKASEEEEE DTQQIDHTEI [ 960]

#China-CG12 KIETAANETA GEAEKAATFA KEAADAAKDT NEAVTLAVAK EKVEKALKAA KEAKKANEKA SYALIRTKKQ YALEPLEITS EAGYNITEKE EQVKEEIEEQ DDKASEEEEE DTLQIDNTEV [ 960]

#China-CG8 KIETAANETA GEAEKAATFA KEAADAAKDT NEAVTLAVAK EKVEKALKAA KEAKKANEKA SYALIRTKKQ HALEPLEITS EAGYNITEKE EQVKEEIEEQ EDKASEEEEE DTLQTDQTEI [ 960]

#AY454084-Bangl KIETEANATA GEAQKAATFA KEAADAAKDT NEAVTLAVAK EKVEKALKAA KEAKKANEKA SYALIRTKKQ HALEPLEITS EAGYNITEKE EQVKEEIEEQ EDKANEEEDE DTQQTDHTEV [ 960]

#AY454097-SL57 KIETEANATA GEAQKAATFA KEAADAAKDT NEAVTLAVAK EKVEKALKAA KEAKKASEKA SYALIRTKKQ HALEPLEITS EAGYNITEKE EQVKEEIEEQ EDKASEEEEE DTHQTDQTEI [ 960]

#AY454096-SL KIETEANATA GEAQKAATFA KEAADAAKDT NEAVTLAVAK EKVEKALKAA KEAKKASEKA SYALIRTKKQ HALEPLEITS EAGYNITEKE EQVKEEIEEQ EDKASEEEEE DTHQTDQTEI [ 960]

#Thai-106 KIETEANAAA GEAQKAATFA KEAADAAKDT NEAVTLAVAK EKVEKALRAA KEAKKASEKA SYPLIRTKKQ YALEPLEITS EAGYNITEKE EQVKEEIEEQ EDKASEEEEE DTLQTDQTEI [ 960]

#Thai-NR1 KIETEANATA GEAQKAATFA KEAADAAKDT NEAVTLAVAK EKVEKALKAA KEAKKASEKA SYALIRTKKQ YALEPLEITS EAGYNITEKE EQVKEEIEEQ EDKANEEEDE DTQQTDHTEV [ 960]

#Thai-T107 KIETEANATA GEAQKAATFA KEAADAAKDT NEAVTLAVAK EKVEKALKAA KEAKKASEKA SYALIRTKKQ YALEPLEITS EAGYNITEKE EQVKEEIEEQ EDKANEEEDE DTQQTDHTEV [ 960]

#Thai-113 KIETEANATA GEAQKAATFA KEAADAAKDT NEAVTLAVAK EKVEKALKAA KEAKKASEKA SYALIRTKKQ YALEPLEITS EAGYNITEKE EQVKEEIEEQ EDKANEEEDE DTQQTDHTEV [ 960]

#Thai-430 KIETEANATA GEAQKAATFA KEAADAAKDT NEAVTLAVAK EKVEKALKAA KEAKKASEKA SYALIRTKKQ HALEPLEITS EAGYNITEKE EQVKEEIEEQ EDKASEEEEE DTQQIDNTEV [ 960]

#China-CG11 KIETEANATA GEAQKAATFA KEAADAAKDT NEAVTLAVAK EKVEKALKAA KEAKKASEKA SYALIRTKKQ HALEPLEITS EAGYNITEKE EQVKEEIEEQ EDKASEEEEE DTHQTDQTEI [ 960]

#AY454083-Chess KIETVANETA GEAEKAATFA KEAADAAKDT NEAVTLAVAK EKVEKALKAA KEAKKANEKA SYALIRTKKQ YALEPLEITS EAGYNITEKE EQVKEEIEEQ DDKASEEEEE DTHQTDQTEI [ 960]

#Thai-TC103 KIETEANATA GEAQKAATFA KEAADAAKDT NEAVTLAVAK EKVEKALKAA KEAKKANEKA SYALIRTKKQ YALEPLEITS EAGYNITEKE EQVKEEIEEQ DDKASEEEEE DTHQTDQTEI [ 960]

#Thai-TV400 KIETEANATA GEAQKAATFA KEAADAAKDT NEAVTLAVAK EKVEKALKAA KEAKKANEKA SYALIRTKKQ HALEPLEITS EAGYNITEKE EQVKEEIEEQ EDKASEEEEE DTHQTDQTEI [ 960]

#AY454089-Br781T KIETAANETA GEAEKAATFA KEAADAAKDT NEAVTLAVAK EKVEKALKAA KEAKKANEKA SYALIRTKKQ YALEPLEITS EAGYNITEKE EQVKEEIEEQ DDKASEEEEE DTLQIDNTEV [ 960]

#PVNG01493-NK KIETAANETA GEAEKAATFA KEAADAAKDT NEAVTLAVAK EKVEKALKAA KEAKKANEKA SYALIRTKKQ YALEPLEITS EAGYNITEKE EQVKEEIEEQ EDKASEEEEE DTLQTDQTEI [ 960]

#Thai-411 KIETAANETV GEAQKAATFA KEAADAAKDT NEAVTLAVAK EKVEKALKAA KEAKKANEKA SYALIRTKKQ HALEPLEITS EAGYNITEKE EQVKEEIEEQ EDKASEEEEE DTQQIDNTEV [ 960]

#Thai-TF83 KIETAANETA GEAEKAATFA KEAADAAKDT NEAVTLAVAK EKVEKALKAA KEAKKANEKA SYALIRTKKQ YALEPLEITS EAGYNITEKE EQVKEEIEEQ KDKASEEEEE DTLQTDQTEI [ 960]

#Thai-109 KIETAANETA GKAEKAATFA KEAADAAKDT NEAVTLAVAK EKVEKALKAA KEAKKANEKA SYALIRTKKQ YALEPLEITS EAGYNITEKE EQVKEEIEEQ EDKASEEEEE DTLQIDNTEV [ 960]

#Thai-410 KIETAANETA GEAEKAATFA KEAADAAKDT NEAVTLAVAK EKVEKALKAA KEAKKANEKA SYALIRTKKQ YALEPLEITS EAGYNITEKE EQVKEEIEEQ KDKASEEEEE DTLQTDQTEI [ 960]

#AY454098-Thai KIETAANETA GEAEKAATFA KEAADAAKDT NEAVTLAVAK EKVEKALKAA KEAKKANEKA SYALIRTKKQ YALEPLEITS EAGYNITEKE EQVKEEIEEQ EDKASEEEEE DTLQTDQTEI [ 960]

#AF099662-Belem KIETEANATA GEAQKAAAFA KEAADAAKDT NEAVTLAVAK EKVEKALKAA KEAKKANEKA SYALIRTKKQ YALEPLEITS EAGYNITEKE EQVKEEIEEQ DDKASEEEEE DTHQTDQTEI [ 960]

#AY454080-Br1B KIETEANATA GEAQKAAAFA KEAADAAKDT NEAVTLAVAK EKVEKALKAA KEAKKANEKA SYALIRTKKQ YALEPLEITS EAGYNITEKE EQVKEEIEEQ DDKASEEEEE DTHQTDQTEI [ 960]

#AY454085-Br56 KIETEANATA GEAQKAATFA KEAADAAKDT NEAVTLAVAK EKVEKALKAA KEAKKANEKA SYALIRTKKQ HALEPLEITS EAGYKITEKE EQVKEEIEEQ EDKASEEEEE DTHQTDQTEI [ 960]

#Thai-115 KIETEANATA GEAQKAAAFA KEAADAAKDT NEAVTLAVAK EKVEKALKAA KEAKKANEKA SYALIRTKKQ YALEPLEITS EAGYNITEKE EQVKEEVEEQ EDKASEEEEE DTLQTDQTEI [ 960]

#Thai-TF91 KIETEANATA GEAQKAAAFA KEAADAAKDT NEAVTLAVAK EKVEKALKAA KEAKKANEKA SYALIRTKKQ YALEPLEITS EAGYNITEKE EQVKEEIEEQ DDKASEEEEE YTHQTDQTEI [ 960]

#Thai-126 KIETAANETA GEAERAATFA KEAADAAKDT NEAVTLAVAK EKVEKALKAA KEAKKANEKA SYALIRTKKQ YALEPLEITS EAGYNITEKE EQVKEEIEEQ DDKASEEEEE DTLQIDNTEV [ 960]

#Thai-442 KIETEANATA GEAQKAAAFA KEAADAAKDT NEAVTLAVAK EKVEKALKAA KEAKKANEKA SYALIRTKKQ YALEPLEITS EAGYNITEKE GQVKEEIEEQ DDKASEEEEE DTHQTDQTEI [ 960]

#Thai-408 KIETEANATA GEAQKAAAFA KEAADAAKDT NEAVTLAVAK EKVEKALKAA KEAKKANEKA SYALIRTKKQ YALEPLEITS EAGYNITEKE EQVEEEIEEQ DDKASEEEEE DTQQIDHTEI [ 960]

#India-IN1 KIETEANATA GEAQKAAAFA KEAADAAKDT NEAVTLAVAK EKVEKALKAA KEAKKANEKA SYALIRTKKQ YALEPLEITS EAGYNITEKE EQVKEEIEEQ DDKASEEEEE DTHQTDQTEI [ 960]

#Thai-TB123 KIETAANETA GEAEKAATFA KEAADAAKDT NEAVTLAVAK EKVEKALKAA KEAKKANEKA SYALIRTKKQ YALEPLEITS EAGYNITEKE EQVKEEIEEQ DDKASEEEEE DTLQIDNTEV [ 960]

#Thai-T139 KIETEANATA GEAQKAATFA KEAADAAKDT NEAVTLAVAK EKVEKALKAA KEAKKASEKA SYALIRTKKQ YALEPLEITS EAGYNITEKE EQVKEEIEEQ EDKANEEEDE DTQQTDHTEV [ 960]

#Thai-TF127 KIETAANETA GEAEKAATFA KEAADAAKDT NEAVTLAVAK EKVEKALKAA KEAKKANEKA SYALIRTKKQ YALEPLEITS EAGYNITEKE EQVKEEIEEQ EDKANEEEDE DTQQTDHTEV [ 960]

#Thai-104 KIETAANETV GEAQKAATFA KEAADAAKDT NEAVTLAVAK EKVEKALKAA KEAKKANEKA SYALIRTKKQ YALEPLEITS EAGYNITEKE EQVKEEIEEQ DDKASEEEEE DTLQIDNTEV [ 960]

#Thai-413 KIETAANETA GEAEKAATFA KEAADAAKDT NEAVTLAVAK EKVEKALKAA KEAKKANEKA SYALIRTKKQ HALEPLEITS EAGYNITEKE EQVKEEIEEQ EDKASEEEEE DTLQTDQTEI [ 960]

#Thai-431 KLETAANETA GEAGKAATFA KEAADAAKDT NEAVTLAVAK EKVEKALKAA KEAKKANEKA SYALIRTKKQ HALEPLEITS EAGYNITEKE EQVKEEIEEQ EDKASEEEEE DTQQIDNTEV [ 960]

#Thai-447 KIETAANETV GEAQEAATFA KEAADAAKDS NEAVTLAVAE EKVEKALKAA KEAKKANEKA SYALIRTKKQ HALEPLEITS EAGYNITEKE EQVKEEIEEQ EDKASEEEEE DTQQIDNTEV [ 960]

#Thai-465 KIETAANETV GEAQEAATFA KEAADAAKDS NEAVTLAVAE EKVEKALKAA KEAKKANEKA SYALIRTKKQ YALEPLEITS EAGYNITEKE EQVKEEIEEQ DDKASEEEEE DTQQIDHTEV [ 960]

#Thai-425 KIETAANETA GEAEKAATFA KEAADAAKDT NEAVTLAVAK EKVEKALKAA KEAKKANEKA SYALIRTKKQ YALEPLEITS EAGYNITEKE EQVKEEIEEQ EDKANEEEDE DTQQTDHTEV [ 960]

#Thai-421 KIETEANATA GEAQKAATFA KEAADAAKDT NEAVTLAVAK EKVEKALKAA KEAKKASEKA SYALIRTKKQ YALEPLEITS EAGYNISEKE EQVKEEIEEQ EDKASEEEEE DTLQTDQTEI [ 960]

#Papau-PNN1 KIETEANATA GEAQKAATFA KEAADAAKDT NEAVTLAVAK EKVEKALKAA KEAKKANEKA SYALIRTKKQ HALEPLEITS EAGYNITEKE EQVKEEIEEQ EDKASEEEEE DTHQTDQTEI [ 960]

#China-CG15 KIETEANATA GEAQKAAAFA KEAADAAKDT NEAVTLAVAK EKVEKALKAA KEAKKANEKA SYALIRTKKQ YALEPLEITS EAGYNITEKE EQVKEEIEEQ DDKASEEEEE DTHQTAQTEI [ 960]

#AY454087-Br67T KIETEANATA GEAQKAATFA KEAADAAKDT NEAVTLAVAK EKVEKALKAA KEAKKANEKA SYALIRTKKQ HALEPLEITS EAGYKITEKE EQVKEEIEEQ EDKASEEEEE DTHQTDQTEI [ 960]

#AY454088-Br781B KIETAANETA GEAEKAATFA KEAADAAKDT NEAVTLAVAK EKVEKALKAA KEAKKANEKA SYALIRTKKQ YALEPLEITS EAGYNITEKE EQVKEEIEEQ DDKASEEEEE DTQQIDQTQI [ 960]

#AY454090-EcuB KIETEANATA GEAQKAAAFA KEAADAAKDT NEAVTLAVAK EKVEKALKAA KEAKKANEKA SYALIRTKKQ YALEPLEITS EAGYNITEKE EQVKEEIEEQ DDKASEEEEE DTHQTDQTEI [ 960]

#AY454094-Ong KIETEANATA GEAQKAATFA KEAADAAKDT NEAVTLAVAK EKVEKALKAA KEAKKASEKA SYALIRTKKQ YALEPLEITS EAGYNITEKE EQVKEEIEEQ DDKASEEEEE DTQQIDHTEI [ 960]

#AY454086-Br67B ---------- ---------- ---------- ---------- ---------- ---------- ---------- ---------- ---------- ---------- ---------- ---------- [ 960]

#Thai-105 KIETAANETV GEAQKAATFA KEAADAAKDT NEAVTLAVAK EKVEKALKAA KEAKKANEKA SYALIRTKKQ HALEPLEITS EAGYNITEKE EQVKEEIEEQ EDKASEEEEE DTLQTDQTEI [ 960]

#PVMG01384-Mauri KIETAANETA GEAEKAATFA KEAADAAKDT NEAVTLAVAK EKVEKALKAA KEAKKANEKA SYALIRTKKQ YALEPLEITS EAGYNITEKE EQVKEEIEEQ KDKASEEEEE DTLQTDQTEI [ 960]

#PVIIG04181-Indi KIETEANATA GEAQKAAAFA KEAADAAKDT NEAVTLAVAK EKVEKALKAA KEAKKANEKA SYALIRTKKQ YALEPLEITS EAGYNITEKE EQVKEEIEEQ DDKASEEEEE DTHQTDQTEI [ 960]

#PVBG05499-Brazi KIETAANETA GEAEKAATFA KEAADAAKDT NEAVTLAVAK EKVEKALKAA KEAKKANEKA SYALIRTKKQ YALEPLEITS EAGYNITEKE EQVKEEIEEQ DDKASEEEEE DTLQIDNTEV [ 960]

Conserved >

#XM001613146-Sal DEVDISVDNE EEEEGAAEEQ IE--GEKDTP TKEAKEEQTS GEKILDDKEA HKTLAEKFKD SNTAKTGGVE FLETLISDVG EDTLKNLQQD LHQYFKGK [1058]

#AY454081-Br1T DEVDISVDNE EEEEGAADEQ SE--GEKDTP EKEAKEEQAS GEQILDDKEA HKTLAEKFKD SNPAKTEGDK ILETLISDVG EDTLKNMQQD LHQYFKGK [1058]

#AY454082-Br69 DEVDISVDNE EEEEGAADEQ SE--GEKDTP EKEAKEEQAS GEQILDDKEA HKTLAEKFKD SNPAKTEGDK ILETLISDVG EDTLKNMQQD LHQYFKGK [1058]

#AY454092-India DEVDISVDNE EEEEGAADEQ SE--GEKDTP EKEAKEEQAS GEQILDDKE* ********** ********** ********** ********** ******** [1058]

#AY454091-EcuT DEVDISVDNE EEEEGASDEQ IE--GKKDTP EKEAKEEQAS GEQILDDKE* ********** ********** ********** ********** ******** [1058]

#Thai-414 DEVDISVDNE EEEEGASDEQ IE--GKKDTP EKEAKEEQAS GEQILDDKEA HKTLAEKFKD SIPAKTEGDK ILETLISDVG EDTLKNLQQD LHQYFKGK [1058]

#Thai-129 DEVDISVDNE EEEEGAADEQ SE--GEKDTP EKEAKEEQAS GEQILDDKEA HKTLAEKFKD SNPAKTEGDK ILETLISDVG EDTLKNMQQD LHQYFKGK [1058]

#Thai-T120 DEVDISVDNE EEEEGASDEQ IE--GKKDTP EKEAKEEQAS GEQILDDKEA HKTLAEKFKD SIPAKTEGDK ILETLISDVG EDTLKNLQQD LHQYFKGK [1058]

#Thai-462 DEVDISVDNE EEEEGAADEQ IE--GKKDTP EKEAKEEQAS GEQILDDKEA HKTLAEKFKD SNPAKTEGDK ILETLISDVG EDTLKNLQQD LHQYFKGK [1058]

#China-CG12 DEVDISVDNE EEEEGASDEQ IE--GKKDTP EKEAKEEQAS GEQILDDKEA HKTLAEKFKD SIPAKTEGDK ILETLISDVG EDTLKNLQQD LHQYFKGK [1058]

#China-CG8 DEVDISVDNE EEEEGAADEQ SE--GEKDTP EKEAKEEQAS GEQILDDKEA HKTLAEKFKD SNPAKTEGDK ILETLISDVG EDTLKNMQQD LHQYFKGK [1058]

#AY454084-Bangl DEVDINVDNE EEEEGTAEEQ AEEEGEKDTP KKEGNEEPAS GEQILDDKE* ********** ********** ********** ********** ******** [1058]

#AY454097-SL57 DEVDISVDNE EEEEGVADEQ IE--GKKDTP EKEAKEEQAS GEQILDDKE* ********** ********** ********** ********** ******** [1058]

#AY454096-SL DEVDISVDNE EEEEGVADEQ IE--GKKDTP EKEAKEEQAS GEQILDDKE* ********** ********** ********** ********** ******** [1058]

#Thai-106 DEVDISVDNE EEEEGAADEQ SE--GEKDTP EKEAKEEQAS GEQILDDKEA HKTLAEKFKD SNPAKTEGDK ILETLISDVG EDTLKNMQQD LHQYFKGK [1058]

#Thai-NR1 DEVDINVDNE EEEEGTAEEQ AEEEGEKDTP KKEGNEEPAS GEQILDDKEA HKTLAEKFKD SNPAKTEGDK ILETLISDVG EDTLKNLQQD LHQYFKGK [1058]

#Thai-T107 DEVDINVDNE EEEEGTAEEQ AEEEGEKDTP KKEGNEEPAS GEQILDDKEA HKTLAEKFKD SNPAKTEGDK ILETLISDVG EDTLKNLQQD LHQYFKGK [1058]

#Thai-113 DEVDINVDNE EEEEGTAEEQ AEEEGEKDTP KKEGNEEPAS GEQILDDKEA HKTLAEKFKD SNPAKTEGDK ILETLISDVG EDTLKNMQQD LHQYFKGK [1058]

#Thai-430 DEVDISVDNE EEEEGTAEEQ IE--GEKDTT EKEAKEEPAS GEQILDDKEA HKTLAEKFKD SNPAKTGGVE FLETLISDVG EDTLKNLQQD LHQYFKGK [1058]

#China-CG11 DEVDINVDNE EEEEGTAEEQ AEEEGEKDTP KKEGNEEPAS GEQILDDKEA HKTLAEKFKD SNPAKTEGDK ILETLISDVG EDTLKNLQQD LHQYFKGK [1058]

#AY454083-Chess DEVDISVDNE EEEEGAADEQ SE--GEKDTP EKEAKEEQAS GEQILDDKEA HKTLAEKFKD SNPAKTEGDK ILETLISDVG EDTLKNMQQD LHQYFKGK [1058]

#Thai-TC103 DEVDISVDNE EEEEGTADEQ IE--GEKDTP EKEAKEEPAS GEKILDDKEA HKTLAEKFKD SNPAKTEGDK ILETLISDVG EDTLKNMQQD LHQYFKGK [1058]

#Thai-TV400 DEVDISVDNE EEEEGVADEQ IE--GKKDTP EKEAKEEQAS GEQILDDKEA HKTLAEKFKD SNPAKTEGDK ILETLISDVG EDTLKNLQQD LHQYFKGK [1058]

#AY454089-Br781T DEVDISVDNE EEEEGASDEQ IE--GKKDTP EKEAKEEQAS GEQILDDKE* ********** ********** ********** ********** ******** [1058]

#PVNG01493-NK DEVDISVDNE EEEEGAADEQ SE--GEKDTP EKEAKEEQAS GEQILDDKEA HKTLAEKFKD SNPAKTEGDK ILETLISDVG EDTLKNLQQD LHQYFKGK [1058]

#Thai-411 DEVDISVDNE EEEEGTAEEQ IE--GEKDTT EKEAKEEPAS GEQILDDKEA HKTLAEKFKD SNPAKTEGDK ILETLISDVG EDTLKNMQQD LHQYFKGK [1058]

#Thai-TF83 DEVDISVDNE EEEEGAADEQ SE--GEKDTP EKEAKEEQAS GEQILDDKEA HKTLAEKFKD SNPAKTGGVE FLETLISDVG EDTLKNLQQD LHQYFKGK [1058]

#Thai-109 DEVDISVDNE EEEEGASDEQ IE--GKKDTP EKEAKEEQAS GEQILDDKEA HKTLAEKFKD SNPAKTEGDK ILETLISDVG EDTLKNLQQD LHQYFKGK [1058]

#Thai-410 DEVDISVDNE EEEEGAADEQ SE--GEKDTP EKEAKEEQAS GEQILDDKEA HKTLAEKFKD SNPAKTGGVE FLETLISDVG EDTLKNLQQD LHQYFKGK [1058]

#AY454098-Thai DEVDISVDNE EEEEGAADEQ SE--GEKDTP EKEAKEEQAS GEQILDDKE* ********** ********** ********** ********** ******** [1058]

#AF099662-Belem DEVDISVDNE EEEEGTADEQ IE--GEKDTP EKEAKEEPAS GEKILDDKEA HKTLAEKFKD SNPAKTEGDK ILETLISDVG EDTLKNMQQD LHQYFKGK [1058]

#AY454080-Br1B DEVDISVDNE EEEEGTADEQ IE--GEKDTP EKEAKEEPAS GEKILDDKEA HKTLAEKFKD SNPAKTEGDK ILETLISDVG EDTLKNLQQD LHQYFKGK [1058]

#AY454085-Br56 DEVDISVDNE EEEEGVADEQ IE--GKKDTP EKEAKEEQAS GEQILDDKE* ********** ********** ********** ********** ******** [1058]

#Thai-115 DEVDISVDNE EEEEGAADEQ SE--GEKDTP EKEAKEEQAS GEQILDDKEA HKTLAEKFKD SNPAKTEGDK ILETLISDVG EDTLKNMQQD SHQYFKGK [1058]

#Thai-TF91 DEVDISVDNE EEEEGTADEQ IE--GEKDTP EKEAKEEPAS GEKILDDKEA HKTLAEKFKD SNPAKTEGDK ILETLISDVG EDTLKNMQQD LHQYFKGK [1058]

#Thai-126 DEVDISVDNE EEEEGASDEQ IE--GKKDTP EKEAKEEQAS GEQILDDKEA HKTLAEKFKD SIPAKTEGDK ILETLISDVG EDTLKNLQQD LHQYFKGK [1058]

#Thai-442 DEVDISVDNE EEEEGTADEQ IE--GEKDTP EKKAKEEQAS GEQILDDKEA HKTLAEKFKD SNPAKTEGDK ILETLISDVG EDTLKNMQQV LHQYFKGK [1058]

#Thai-408 DEVDISVDNE EEEEGAADEQ IE--GKKDTP EKEAKEEPAS GEKILDDKEA HKTLAEKFKD SNPAKTEGDK ILETLISDVG EDTLKNLQQD LHQYFKGK [1058]

#India-IN1 DEVDISVDNE EEEEGTADEQ IE--GEKDTP EKEAKEEPAS GEKILDDKEA HKTLAEKFKD SNPAKTEGDK ILETLISDVG EDTLKNMQQD LHQYFKGK [1058]

#Thai-TB123 DEVDISVDNE EEEEGASDEQ IE--GKKDTP EKEAKEEQAS GEQILDDKEA HKTLAEKFKD SNPAKTEGDK ILETLISDVG EDTLKNLQQD LHQYFKGK [1058]

#Thai-T139 DEVDINVDNE EEEEGTAEEQ AEEEGEKDTP KKEGNEEPAS GEQILDDKEA HKTLAEKFKD SNPAKTEGDK ILETLISDVG EDTLKNLQQD LHQYFKGK [1058]

#Thai-TF127 DEVDINVDNE EEEEGTAEEQ AEEEGEKDTP KKEGNEEPAS GEQILDDKEA HKTLAEKFKD SNPAKTEGDK ILETLISDVG EDTLKNLQQD LHQYFKGK [1058]

#Thai-104 DEVDISVDNE EEEEGASDEQ IE--GKKDTP EKEAKEEQAS GEQILDDKEA HKTLAEKFKD SNPAKTEGDK ILETLISDVG EDTLKNLQQD LHQYFKGK [1058]

#Thai-413 DEVDISVDNE EEEEGASDEQ IE--GEKDTP EKEAKEEQAS GEQILDDKEA HKTLAEKFKD SNPAKTEGDK ILETLISDVG EDTLKNLQQD LHQYFKGK [1058]

#Thai-431 DEVDISVDNE EEEEGTAEEQ IE--GEKDTT EKEAKEEPAS GEQILDDKEA HKTLAEKLKD SNPAKTEGDE ILETLISDVG EDTLKNMQQD LHQYFKGK [1058]

#Thai-447 DEVDISVDNE EEEEGTAEEQ IE--GEKDTT EKEAKEEPAS GEQILDDKEA HKTLAEKFKD SNPAKTEGDK ILETLISDVG EDTLKNMQQD LRQYFKGK [1058]

#Thai-465 DEVDISVDNE EEEEGASDEQ IE--GEKDTP EKEAKEEQAS GEQILDDKEA HKTLAEKFKD SIPAKTEGDK ILETLISDVG EDTLKNMQQD LHQYFKGK [1058]

#Thai-425 DEVDINVDNE EEEEGTAEEQ IE--GEKDTT EKEAKEEPES GEQILDDKEA HKTLAEKFKD SNPAKTEGDK ILETLISDVG EDTLKNMQQD LHQYFKGK [1058]

#Thai-421 DEVDISVDNE EKEEGAADEQ SE--GEKDTP EKEAKEEQAS GEQILDDKEA HKTLAEKFKD SNPAKTEGDK ILGTLISDVG EDTLKNMQQD LHQYFKGK [1058]

#Papau-PNN1 DEVDISVDNE EEEEGVADEQ IE--GKKDTP EKEAKEEQAS GEQILDDKEA HKTLAEKFKD SNPAKTEGDK ILETLISDVG EDTLKNLQQD LHQYFKGK [1058]

#China-CG15 DEVDISVDNE EEEEGAADEQ SE--GEKDTP EKEAKEEQAS GEQILDDKEA HKTLAEKFKD SNTAKTGGVE FLETLISDVG EDTLKNLQQD LHQYFKGK [1058]

#AY454087-Br67T DEVDISVDNE EEEEGVADEQ IE--GKKDTP EKEAKEEQAS GEQILDDKE* ********** ********** ********** ********** ******** [1058]

#AY454088-Br781B DEVDISVDNE EEEEGAAEEQ IE--GEKDTP TKEAKEEQTS GEKILDDKE* ********** ********** ********** ********** ******** [1058]

#AY454090-EcuB DEVDISVDNE EEEEGTADEQ IE--GEKDTP EKEAKEEPAS GEKILDDKE* ********** ********** ********** ********** ******** [1058]

#AY454094-Ong DEVDISVDNE EEEEGAADEQ IE--GKKDTP EKEAKEEQAS GEQILDDKE* ********** ********** ********** ********** ******** [1058]

#AY454086-Br67B ---------- ---------- ---------- -KEAKEEQAS GEQILDDKE* ********** ********** ********** ********** ******** [1058]

#Thai-105 DEVDISVDNE EEEEGAADEQ SE--GEKDTP EKEAKEEQAS GEQILDDKEA HKTLAEKFKD SNPAKTEGDK ILETLISDVG EDTLKNMQQD LHQYFKGK [1058]

#PVMG01384-Mauri DEVDISVDNE EEEEGAADEQ SE---EKDTP EKEAKEEQAS GEQILDDKEA HKTLAEKFKD SNPAKTGGVE FLETLISDVG EDTLKNLQQD LHQYFKGK [1058]

#PVIIG04181-Indi DEVDISVDNE EEEEGTADEQ IE---EKDTP EKEAKEEPAS GEKILDDKEA HKTLAEKFKD SNPAKTGGVE FLETLISDVG EDTLKNLQQD LHQYFKGK [1058]

#PVBG05499-Brazi DEVDISVDNE EEEEGASDEQ IE---KKDTP EKEAKEEQAS GEQILDDKEA HKTLAEKFKD SNPAKTEGDK ILETLISDVG EDTLKNLQQD LHQYFKGK [1058]
