# Supplementary figures and images for: The Plasmodium vivax Merozoite Surface Protein 3β Sequence Reveals Contrasting Parasite Populations in Southern and Northwestern Thailand
Source: PLoS Negl Trop Dis. 2014 Nov 20;8(11):e3336. doi: 10.1371/journal.pntd.0003336 (PMC4238993; doi:10.1371/journal.pntd.0003336)

Figure S2

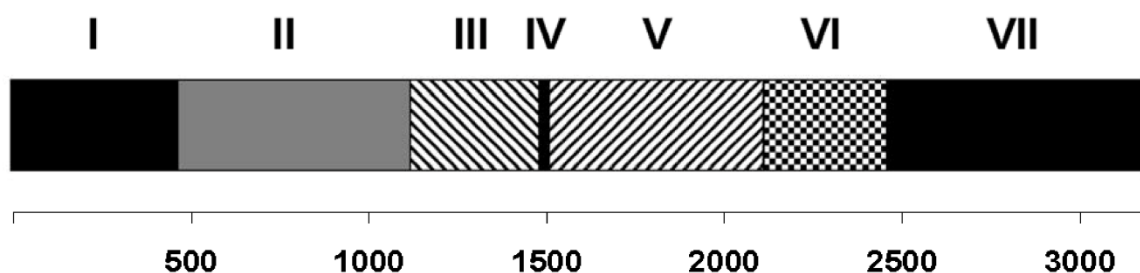

| Breakpoint positions |       |      |
|----------------------|-------|------|
| Event#               | Begin | End  |
| 1                    | 146   | 1177 |
| 2                    | 152   | 966  |
| 3                    | 195   | 329  |
| 4                    | 215   | 550  |
| 5                    | 218   | 1303 |
| 6                    | 242   | 2768 |
| 7                    | 267   | 345  |
| 8                    | 272   | 436  |
| 9                    | 279   | 333  |
| 10                   | 284   | 590  |
| 11                   | 294   | 543  |
| 12                   | 330   | 2020 |
| 13                   | 334   | 551  |
| 14                   | 346   | 868  |
| 15                   | 390   | 1436 |
| 16                   | 440   | 1192 |
| 17                   | 452   | 1709 |
| 18                   | 590   | 893  |
| 19                   | 623   | 2507 |
| 20                   | 628   | 2507 |
| 21                   | 631   | 1764 |
| 22                   | 663   | 868  |
| 23                   | 776   | 868  |
| 24                   | 782   | 1694 |
| 25                   | 869   | 966  |
| 26                   | 963   | 1523 |
| 27                   | 1116  | 2568 |
| 28                   | 1194  | 2239 |
| 29                   | 1434  | 1764 |
| 30                   | 1710  | 2436 |
| 31                   | 1720  | 2354 |
| 32                   | 2018  | 2176 |
| 33                   | 2021  | 2354 |
| 34                   | 2025  | 2222 |
| 35                   | 2126  | 2506 |
| 36                   | 2152  | 2339 |
| 37                   | 2213  | 2507 |
| 38                   | 2340  | 3003 |
| 39                   | 2628  | 3041 |
| 40                   | 2702  | 3024 |

Supplement: Figure S2 — Recombination events and breakpoints in the PvMSP3β locus determined from the RDP4 package. Recombination fragments are shown as bars below the gene scheme depicted after Figure 1. Scale is for aligned nucleotide sites. (PDF) [file pntd.0003336.s002.pdf]
